# Supplementary material for: Low back pain in the Bangladeshi adult population: a cross-sectional national survey
Source: BMJ Open. 2022 Sep 8;12(9):e059192. doi: 10.1136/bmjopen-2021-059192 (PMC9462101; doi:10.1136/bmjopen-2021-059192)
Supplement: Author's manuscript [file bmjopen-2021-059192.draft_revisions.pdf]

BMJ Open is committed to open peer review. As part of this commitment we make the peer review history of every article we publish publicly available.

When an article is published we post the peer reviewers' comments and the authors' responses online. We also post the versions of the paper that were used during peer review. These are the versions that the peer review comments apply to.

The versions of the paper that follow are the versions that were submitted during the peer review process. They are not the versions of record or the final published versions. They should not be cited or distributed as the published version of this manuscript.

BMJ Open is an open access journal and the full, final, typeset and author-corrected version of record of the manuscript is available on our site with no access controls, subscription charges or pay-per-view fees (http://bmjopen.bmj.com).

If you have any questions on BMJ Open's open peer review process please email
info.bmjopen@bmj.com

BMJ Open

Low Back Pain in the Bangladeshi Adult Population: A Cross-sectional National Survey

Journal:	*BMJ Open*
Manuscript ID	bmjopen-2021-059192
Article Type:	Original research
Date Submitted by the Author:	10-Nov-2021
Complete List of Authors:	Momen Majumder, Muhammad Shoaib; Bangabandhu Sheikh Mujib Medical University, Rheumatology<br>Hakim, Ferdous; World Health Organization Bangladesh<br>Bandhan, Iftekhar ; Bangabandhu Sheikh Mujib Medical University, Rheumatology<br>Razzaque, Mohammad Abdur; Chittagong Medical College, Medicine<br>Zahid-Al-Quadir, Ahmad ; Sylhet MAG Osmani Medical College, Medicine<br>Ahmed, Shamim; Bangabandhu Sheikh Mujib Medical University<br>Choudhury, Minhaj; Bangabandhu Sheikh Mujib Medical University, Rheumatology<br>Haq, Syed; Bangabandhu Sheikh Mujib Medical University, Rheumatology<br>Zaman, MM; WHO Bangladesh
Keywords:	EPIDEMIOLOGY, RHEUMATOLOGY, PUBLIC HEALTH

SCHOLARONE™
Manuscripts

I, the Submitting Author has the right to grant and does grant on behalf of all authors of the Work (as defined in the below author licence), an exclusive licence and/or a non-exclusive licence for contributions from authors who are: i) UK Crown employees; ii) where BMJ has agreed a CC-BY licence shall apply, and/or iii) in accordance with the terms applicable for US Federal Government officers or employees acting as part of their official duties; on a worldwide, perpetual, irrevocable, royalty-free basis to BMJ Publishing Group Ltd ("BMJ") its licensees and where the relevant Journal is co-owned by BMJ to the co-owners of the Journal, to publish the Work in this journal and any other BMJ products and to exploit all rights, as set out in our licence.

The Submitting Author accepts and understands that any supply made under these terms is made by BMJ to the Submitting Author unless you are acting as an employee on behalf of your employer or a postgraduate student of an affiliated institution which is paying any applicable article publishing charge ("APC") for Open Access articles. Where the Submitting Author wishes to make the Work available on an Open Access basis (and intends to pay the relevant APC), the terms of reuse of such Open Access shall be governed by a Creative Commons licence – details of these licences and which Creative Commons licence will apply to this Work are set out in our licence referred to above.

Other than as permitted in any relevant BMJ Author's Self Archiving Policies, I confirm this Work has not been accepted for publication elsewhere, is not being considered for publication elsewhere and does not duplicate material already published. I confirm all authors consent to publication of this Work and authorise the granting of this licence.

For peer review only

Low Back Pain in the Bangladeshi Adult Population: A Cross-sectional National Survey

Short Running Head: *Low Back Pain in Bangladesh*

Authors: Muhammad Shoaib Momen Majumder, Ferdous Hakim, Iftekhar Hussain Bandhan, Mohammad Abdur Razzaque, Ahmad Zahid-Al-Quadir, Shamim Ahmed, Minhaj Rahim Choudhury, Syed Atiqul Haq, Mohammad Mostafa Zaman

Author names, highest academic degrees, appointments, Email and ORCID:

Muhammad Shoaib Momen Majumder, FCPS (Medicine), MD (Rheumatology); Department of Rheumatology, Bangabandhu Sheikh Mujib Medical University (Ringgold ID 74464), Bangladesh, Email: momen.shoaib@gmail.com, ORCID ID: https://orcid.org/0000-0002-7901-0847;

Ferdous Hakim, MPH (Epidemiology), Research and Publication, World Health Organization Bangladesh (Ringgold ID 215134), Email: hakimf@who.int, ORCID: https://orcid.org/0000-0003-2376-3978;

Iftekhar Hussain Bandhan, MD (Rheumatology), Department of Rheumatology, Bangabandhu Sheikh Mujib Medical University (Ringgold ID 74464), Bangladesh, Email: ibandhan@gmail.com, ORCID ID: https://orcid.org/0000-0002-3800-6155;

Mohammad Abdur Razzaque, MD (Rheumatology), Register, Department of Medicine, Chittagong Medical College (Ringgold ID 467859), Bangladesh, Email: rhrazzaq@gmail.com, ORCID ID: https://orcid.org/0000-0002-2395-542X;

Ahmad Zahid-Al-Quadir, MD (Rheumatology), Department of Medicine, Sylhet MAG Osmani Medical College (Ringgold ID 469196), Email: drzahidssmc@gmail.com, https://orcid.org/0000-0003-4178-6487;

Shamim Ahmed, FCPS (Medicine), Associate Professor of Rheumatology, Bangabandhu Sheikh Mujib Medical University (Ringgold ID 74464), Dhaka, Bangladesh, Email: dr_shamim_ahmed@yahoo.com;

Minhaj Rahim Choudhury, MD, FCPS (Medicine) Professor of Rheumatology, Bangabandhu Sheikh Mujib Medical University (Ringgold ID 74464), Dhaka, Bangladesh, Email: drminhaj11@gmail.com, ORCID ID: https://orcid.org/0000-0002-8695-5240;

Syed Atiqul Haq, FCPS (Medicine), MD, FRCP, Professor of Rheumatology, Bangabandhu Sheikh Mujib Medical University (Ringgold ID 74464), Dhaka, Bangladesh, Email: haqsyedatiqul@gmail.com, ORCID ID: https://orcid.org/0000-0003-4154-7283

Mohammad Mostafa Zaman, PhD (Epidemiology), Advisor, Research and Publication, World Health Organization Bangladesh (Ringgold ID 215134), Email: zamanm@who.int, ORCID ID: https://orcid.org/0000-0002-1736-1342;

Counts: Number of words (abstract): 248, number of words (body): 2850, number of references: 50, number of figures: 03, number of tables: 03

Name, address, email of the author responsible for correspondence:

For peer review only

DRAFT

For peer review only - http://bmjopen.bmj.com/site/about/guidelines.xhtml

Muhammad Shoaib Momen Majumder, FCPS (Medicine), MD (Rheumatology); Department of Rheumatology, Bangabandhu Sheikh Mujib Medical University (Ringgold ID 74464), Bangladesh, Email: momen.shoaib@gmail.com, ORCID ID: https://orcid.org/0000-0002-7901-0847

ABSTRACT

Objective:

Low back pain (LBP) is a common musculoskeletal disorder. This study aims to determine the residence- and sex-specific prevalence and the risk factors of LBP in Bangladesh.

Methods:

The study subjects (ages ≥18 years) were identified from 20 primary sampling units of the national census following a cross-sectional multi-stage stratified sampling design. We considered the mechanical type of LBP for this study. A Bangla version of the modified Community Oriented Program for Control of Rheumatic Disorders questionnaire was used. A team of trained field workers, rheumatology residents and rheumatologists collected the data.

Results:

Two thousand subjects were approached, but 1843 could be screened. Among them, 561 had musculoskeletal disorders, and 343 were diagnosed with LBP. The age-adjusted prevalence of LBP was 19.4% (95% confidence interval, CI: 14.0–24.8), which was higher in women (27.2%, 19.3-35.1) than men (14.0%, 8.7-19.3). The prevalence persistently increased from age group 18-34 years (12.8%, 8.5-17.2) to ≥55 years (23.5%, 16.0-31.0). People with no education had the highest prevalence (35.0%, 25.4-44.6). The prevalences did not differ between urban and rural residential locations. Four factors were significantly associated with LBP: age (adjusted odds ratio: 2.5, 95% CI: 1.8–3.4), female sex (2.4, 1.8–3.2), absence of formal education (1.5, 1.1–1.9), and history of physical trauma (1.9, 1.3–2.8).

Conclusion:

LBP is a common problem in Bangladeshi adults. The risk factors are age, female sex, no formal education, and history of physical trauma. These should be addressed adequately to prevent and treat LBP.

Keywords: Low back pain, risk factors, Bangladesh, prevalence, cross-sectional survey

KEY MESSAGES:

What is already known?

- Low back pain–a common musculoskeletal disorder–is an important cause of disability and work loss globally particularly for the lower middle income countries.

What does this study add?

- This study was the first national-level study on musculoskeletal disorders using the primary sampling units of the Bangladesh Bureau of Statistics
- We report here the prevalence of low back pain in Bangladesh segregated by sociodemographic factors, and risk factors.
- Four risk factors were identified for LBP: those were age, female sex, absence of formal education, and history of trauma

What is the impact on future clinical practice?

- Appropriate clinical services, health education and risk reduction strategies can reduce the burden of low back pain.

For peer review only - http://bmjopen.bmj.com/site/about/guidelines.xhtml

INTRODUCTION

Low back pain (LBP) is one of the most frequent medical problems globally. It is defined as pain, stiffness, or muscle tension localized below the costal margin and above the inferior gluteal folds[1]. Up to 84% of adults suffer from LBP at some point in life[2]. The prevalence of chronic LBP is about 23%[3]. Around 11–12% of the population become disabled due to LBP[4]. It causes substantial personal, social and financial burdens globally[4]. In the USA, LBP is the second most frequent cause for a physician consultation[5]. LBP is ranked globally as the topmost cause of disability as it affects mostly working-age people[6]. It accounted for 60.1 million disability-adjusted life-years in 2015. There was a significant increase of LBP by 54% since 1990, and the highest escalation took place in the low and middle-income countries (LMICs)[7]. Disability from LBP is a primary concern for the LMICs, where manual labour is common in an unconventional way. The scope for job switching is restricted in resource constraint countries.

LBP has multi-sectorial health outcomes like a lower quality of life, poorer self-reported health, depression and more workspace absenteeism[8]. As a result, LBP has become an important cause of sick leave and early retirement among the working population[9]. In the USA, approximately 149 million workdays are lost due to LBP, leading to an estimated loss of 100–200 billion US dollars per year[10]. Non-specific LBP is the commonest of all causes of LBP. Non-specific LBP is defined as LBP not particularly associated with severe or particular aetiology like malignancy, infection, fracture, inflammatory condition, radiculopathy or cauda equina syndrome[3].

Albeit high in most studies, there is a difference in LBP prevalence in various epidemiological studies. The estimated prevalence was 84% in Canada, 70% in Denmark, 59% in the UK, and 29% in Iran[11]. The estimated prevalence of LBP in India ranged between 42 and 83%[12 13]. A recent cross-sectional, community-based, epidemiological study conducted in Northern India yielded an estimated lifetime prevalence of 47% in man 57% in women[14]. A Community Oriented Program for Control of Rheumatic Disorders (COPCORD) survey in Bangladesh published in 2005 showed 6.6%, 9.9%, and 9.2% prevalence of LBP in the rural, urban slum, and affluent urban areas, respectively[15]. A cross-sectional national study in Bangladesh in 2015 showed LBP was the top-ranking musculoskeletal disorder (MSD) with a prevalence of 18.6%[16]. This communication focuses more deeply on the

For peer review only - http://bmjopen.bmj.com/site/about/guidelines.xhtml

segregated prevalence, risk factors, disability and work loss due to LBP utilizing data generated by the 2015 study.

METHODS

A detailed description of the methodology is beyond the scope of this article and is described elsewhere[16]. Adults aged 18 years or more comprised the study population through a household level multi-stage stratified cross-sectional survey. The sampling frame was based on the 2001 Bangladesh Census[17]. With a design effect of 1.5 and 85% response rate for four reporting domains (man-woman, urban-rural), the calculated sample size was 1,978, which was rounded to 2,000. It was stratified into seven divisions of rural (Mauza) and urban (Mahalla) areas. Twenty (8 urban and 12 rural) primary sampling units (PSUs) were selected. The first 100 households were consecutively included from each PSU, where even numbers were assigned as man and odd numbers as woman households. In each household, the single respondent was identified from a list of eligible household members with the help of a Kish table. (**Figure 1**)

A detailed manual was prepared before the training for this survey and was used by all field staff. All investigators and the WHO technical team coordinated and conducted the training. The modified COPCORD questionnaire was the survey tool[18]. The English version of the first part of the questionnaire was translated to Bangla, then adapted, validated, and administered by the interviewers. Data were collected for six days from each PSU. There were two recall visits to ensure participation. The research physician interviewed the suspected respondents for MSDs. Opinions were taken from the division level investigator for the doubtful cases. To validate the diagnoses, the investigators made at least one visit to PSUs in their respective divisions.

LBP group of disorders were operationally defined as mechanical type back pain that included non-specific LBP and lumbar spondylosis. Considering the limitation of differentiating investigation in the field, we did not classify LBP beyond this. LBP duration was classified into three groups: acute: up to 6 weeks, subacute: 6-12 weeks, and chronic that persists beyond 12 weeks[19]. Respondents with pain in the muscles, bones, joints, or any part of the body (musculoskeletal symptom) during the preceding seven days or on pain medication with no pain were considered as positive respondents. The research physicians interviewed and thoroughly examined all 'positive' respondents. Data on physical activity

For peer review only - http://bmjopen.bmj.com/site/about/guidelines.xhtml

were calculated into metabolic equivalent tasks (MET)-minutes per week using the STEPwise Surveillance of noncommunicable disease risk factors (STEPS) protocol and divided into quartiles[20]. The 4th quartile was labelled as strenuous physical activity.

The study participants were divided into three subgroups as per age in years: 18-34, 35-54 and 55-99. We considered ownership of household asset items (electricity, television, refrigerator, etc.) for constructing wealth index. In addition, the type of main material used for the roof of the main house (cement, tin and katcha such as bamboo/thatched/straw/gunny etc.) was also included in the model. A principal component analysis was used to create standardized factor scores for each of the items. The total scores for the respondents were calculated and categorised those into quartiles for description from one (lowest household wealth) to four (highest household wealth)[16].

A validated Bangla version of the Bangla Health Assessment Questionnaire - Disability Index (HAQ-DI) was used for the disability score. For determining work loss, the recall period was 12 months[21]. Diabetes was defined as random blood glucose ≥11.1 mmol/l or the use of anti-diabetic medications. Obesity was defined as a body mass index of ≥25 kg/m^2 [22]. Abdominal obesity was defined if the waist circumference ≥90 cm in man, ≥80 cm in the woman[23].

Statistical analysis:

The survey data were entered in and cleaned using Microsoft Excel. However, statistical analysis was done using Epi Info Version 7.1.5.2. Continuous variables were categorised before analysis as appropriate. We estimated the prevalence of LBP with 95% confidence intervals. The prevalence was segregated by residence (urban/ rural) and sex (man/ woman). Nonparametric test (Kruskal-Wallis H test) were used to analyse data that were not normally distributed. Whenever we encountered an unweighted respondent size of less than 25, the confidence intervals were suppressed. Age adjustment of prevalence estimates was made based on the WHO World Population 2000-2025[24]. Factors were checked for association with LBP by comparing LBP with no MSD through 2x2 tables. Univariate logistic regression analysis was done to obtain unadjusted odds ratios. All statistically significant relationships ($P<0.05$) were entered into a model for logistic regression analysis. The adjusted odds ratios and their 95% confidence limits were calculated to identify the strength of association of LBP factors. A detailed description of categorisation and analysis of other variables was described elsewhere[16].

For peer review only - http://bmjopen.bmj.com/site/about/guidelines.xhtml

Ethics Approval and Consent to Participate:

Ethical guidelines, as outlined by the Declaration of Helsinki, were followed throughout the study[25]. Ethical clearance was obtained from the Institutional Review Board of Bangabandhu Sheikh Mujib Medical University (ID 1100). Consent was obtained from the respondents in Bangla as per Institutional Review Board's guidelines.

Patient and Public Involvement:

Patient or the public were not involved in the design, or conduct, or reporting, or dissemination plans of this study.

RESULTS

Characteristics of respondents:

In this nationally representative study, 2,000 adults 18 years or older were approached, and 1,843 (92.2%) agreed to participate[16]. The mean age of the participants was 40.5 (standard deviation 14.7) years, and 51.6% were women. A total of 561 (30.4%) had some type of MSDs. LBP was the most common among MSDs (18.6%, unadjusted), followed by knee osteoarthritis (7.3%) and soft-tissue rheumatism (5.2%). Among the inflammatory rheumatic diseases, the common conditions were rheumatoid arthritis (1.6%) and spondyloarthritis (1.3%).

Characteristics of respondents with LBP:

Background characteristics:

Among the LBP respondents (n=343), 63.3% were women, and 65.3% were from rural areas. Mean age in years (standard deviation) was 44.2 (13.8) overall, and 48.8 (13.0) in men and 41.6 (13.6) in women. The study participants were divided into three subgroups as per age, and the highest number of LBP was observed in the 35–54 age group (n=168). More than half (52.2%, n=180) were homemakers (all women), while the rest constituted other occupations like laborer, business professional, service holder and others. Almost half of the participants with LBP had no formal education (n=150, 43.7%). Overall, according to the wealth index, 30.0% of respondents belonged to the 1st quartile (lowest socioeconomic status). Above one third (36.4%) of the respondents with LBP had additional concomitant MSDs. About one-fourth (23.0%) were overweight and 31.5% had abdominal obesity. **(Table 1)**

26.6 (20.1–33.1)

For peer review only - http://bmjopen.bmj.com/site/about/guidelines.xhtml

Prevalence:

We report here (**Table 2**) the age-adjusted prevalence of LBP to be 19.4% (95% CI: 14.0–24.8), which is significantly higher in women (23.5%, 16.0–31.0) than men (14.0%, 8.7–19.3). There has been a persistent increase in prevalence from 12.8% (95% CI 8.5–17.1) in 18–34 years age group to 23.5% (95% CI 16.0–31.0) in 55–99 years age group. This trend was more prominent in women. The prevalence did not vary significantly among occupational groups. People with no formal education had significantly highest prevalence of LBP (26.6%, 20.1–33.1) compared to other educational groups. Although the highest prevalence (21.6%) was observed in the 1st quartile of the wealth index, it did not vary significantly. LBP was not significantly associated with strenuous physical activity in our sample. We checked LBP prevalence by urban (16.6%, 11.6–21.6) and rural (19.9%, 12.6–27.1) categories, but it did not differ significantly. Among the co-morbidities, the prevalence of LBP was higher among patients of hypertension (23.9%) and obesity (20.6%). The highest prevalence of LBP (81.7%, 74.1–89.3) was seen in 153 respondents who had multiple (two or more) MSDs. **(Figure 2)**

Disability and work loss:

The distribution of Bengali HAQ-DI scores was not normally distributed among the LBP patients. The scores ranged from 0–2.6. The LBP group's median (interquartile range) was 0.6 (0.3–0.9). The difference of Bangla HAQ-DI score between LBP (n=343) and no MSD (n=60) is statistically significant (P<0.0001) by the Kruskal-Wallis H test, indicating that LBP is associated with a higher disability. The distribution of days lost from work for LBP group had a highly skewed distribution and ranged from 0–365 days. However, the difference of days lost from work (n=1,625) between LBP (n=343) and no MSD (n=1,282) is statistically significant (P<0.0001) by the Kruskal-Wallis H test, indicating that LBP is associated with more days lost. **(Figure 3)**

Risk factors:

Univariate logistic regression analysis did not observe any significant association of occupation, wealth indices, tobacco use, obesity, and diabetes (**Table 3**). A significant association was observed for age groups 35–54 and 55–99 years, female sex, lack of education, history of physical trauma, and hypertension according to the unadjusted odds ratio and their 95% confidence intervals (P<0.001). These significant associations persisted in

For peer review only - http://bmjopen.bmj.com/site/about/guidelines.xhtml

the multiple logistic regression analysis having all these variables into the model simultaneously (P<0.001) except for hypertension.

DISCUSSION

LBP is a common medical problem with very high personal and societal impact, leading to poor quality of life and workability[26]. This survey was the first national study with a representative sample where we report that one in five adults in Bangladesh suffers from LBP. The prevalence in Bangladesh is similar to Kuwait (22.7%)[27] and Qatar (23.3%)[28] but lower than northern India (32%)[29] and Iran (29.3%)[11]. Malaysia (11.6%)[30] has a lower prevalence of LBP than we report here. A systematic review of 165 studies from 54 countries revealed the global prevalence of LBP of 12-33%[4]. According to the systematic analysis of the Global Burden of Disease Study 2017, LBP was the leading cause of YLDs (years lived with disability)[31]. In our study, the rural people had a higher prevalence of LBP than the urban people. This difference–though not statistically significant–was probably due to lower doctor concentration in the rural areas, financial limitations, and less education status. However, regional variation was observed in other studies[11].

Age was a risk factor for LBP in this study. The prevalence of LBP persistently increased with age but this was not statistically significant. However an analysis reported that the risk and prevalence of low back pain increased with age[5]. A systematic review of the global prevalence of LBP revealed the association of age was highest in the 40-49 age group[4 32]. The overall prevalence rises with age 65, which gradually reduces thereafter[33]. Some possible explanations are LBP characteristics in older adults that differ from the middle-aged population (less intense back pain, more leg pain, and more depression)[34]. Our study showed a higher prevalence of LBP among women than men, consistent with some other analyses. This could be due to more household or domestic activities among women compared to men. This finding agrees with the results from the national health survey on the Iranian population[11]. Some studies and systematic reviews demonstrated a higher incidence of LBP in women than men[4 32]. Edward *et al.* 2018 found no significant difference in age and sex scores in their study[35]. Higher women prevalence can be partially explained that they have a lower pain threshold than men[36]. The sex differences may be implied with gonadal steroid hormones like estradiol and testosterone that modulate sensitivity to pain and analgesia[37].

For peer review only - http://bmjopen.bmj.com/site/about/guidelines.xhtml

Women always experienced a higher frequency of visceral pain (e.g., during menstruation, pregnancy) than men[38]. It seems that in painful conditions, women exhibit a greater prevalence than men as women report more pain[39].

Leboeuf-Yde considered body weight as a possible weak indicator for LBP in his systematic literature review due to lack of evidence[40]. The results obtained in our study did not demonstrate a statistically significant association between LBP and higher BMI. Some other studies found obesity or high BMI associated with increased risk LBP development and severity[11 41]. In our research, we found that the absence of formal education is significantly associated with LBP. A cross-sectional study in the United States found that LBP is more common in people who have had less than high school education[42]. Other studies in the USA, UK, and Iran found lower educational status had an increased association with LBP and found higher education inversely associated with LBP[11 43]. Several proposed mechanisms may account for the relationship between low academic status and back pain. The amount of formal education contributes to the types of jobs that an individual may involve in, and subsequently, the types of jobs influence LBP[44]. Moreover, health education regarding posture management, lifestyle changes, physical exercises, stress management poorly reached among people with an absence of formal education. We didn't find any significant association of LBP with occupation. In a US-based study, LBP was significantly related to occupational factors such as truck driving, lifting, carrying, pulling, pushing, twisting, and non-driving vibrational exposure[45]. In some European countries, workers involved in heavy weightlifting (≥25 kg) suffered more from LBP[46].

Studies reported associations between LBP and lower social class[11 42] but we did not find any significant association of LBP with economic status. In our analysis, we found there was a significant positive relation between LBP with trauma. Chronic pain following trauma is possible after physical injury. Persistence back pain was more associated with psychological factors like stress, low education status, etc., than the physical injury itself [47]. Lack of formal education was a significant association in this study which may contribute to this factor. LBP may cause inactivity and lack of exercise resulting in weight gain, subsequently creating or exaggerating co-morbid conditions like hypertension and diabetes mellitus[48]. We found no relationship between hypertension and LBP. In a Korean survey, the lifetime prevalence of LBP was 34.4% among the hypertensive respondents, but the adjusted OR of LBP prevalence

For peer review only - http://bmjopen.bmj.com/site/about/guidelines.xhtml

was significantly lower than the normotensive subjects (fully adjusted 0.79, 95% CI 0.70-0.90)[49]. There was no association between diabetes and LBP in this study.

Strengths and Limitations

This is the first nationally representative survey on LBP in Bangladesh, and probably, among all south Asian nations. The field team included rheumatology residents to make valid diagnoses. Most diagnoses were double-checked in the field by the investigators, who are experienced rheumatologists themselves[16]. Despite the fact that their involvement in the field operation, some diagnoses of evolving rheumatological conditions may suffer from sufficient validity because of a lack of adequate quality imaging facilities in rural areas. The sample size calculation of our study was based on combined MSD prevalence[16]. Therefore, a cautious interpretation of the results is necessary because of inadequate sample size, especially when split into reporting domains.

CONCLUSION

This nationally representative study is the first to report the prevalence of LBP by sociodemographic background, comorbidities and risk factors in Bangladesh. One in five adults suffer from LBP. Education and trauma are modifiable risk factors that warrant intervention. These can be addressed through appropriate health education and clinical services.

DRAFT
For peer review only

Abbreviations and acronyms:

BMI	Body mass index
BSMMU	Bangabandhu Sheikh Mujib Medical University
CI	Confidence interval
COPCORD	Community oriented program from control of rheumatic diseases
HAQ-DI	Health Assessment Questionnaire - Disability Index
LBP	Low back pain
MSK	Musculoskeletal
MSD	Musculoskeletal disorder
OR	Odds ratio
PPS	Population proportion to size
PSU	Primary sampling unit
WHO	World health organisation

Acknowledgement: We gratefully acknowledge the field team, divisional coordinators, civil surgeons, upazila health and family planning officers and health assistants for their support.

Author contribution:

Muhammad Shoaib Momen Majumder (MSMM): Conceptualised the area of work, and prepared the first draft of the manuscript and revised the subsequest drafts.

Ferdous Hakim (FH): analysed the data, guided preparation of graphs and tables, interpreted the results, revised the draft manuscript, and coordinated the writing exercise.

Iftekhar Hussain Bandhan (IHB): prepared graphs and tables, revised the draft manuscript.

Mohammad Abdur Razzaque (MAR): reviewed the manuscript and with special reference to the literature review.

Ahmed Zahid-Al-Quadir and Shamim Ahmed: Prepared the training manual, trained the field team, executed the field operation in coordination with the divisional investigators;

Minhaj R Choudhury, Syed Atiqul Haq and Mohammad Mostafa Zaman: Designed the study, guided the analysis and manuscript writing, critically reviewed the results and manuscript drafts, ensured integrity of data.

All authors: Approved publication.

For peer review only
DRAFT

For peer review only - http://bmjopen.bmj.com/site/about/guidelines.xhtml

Funding: Preparing this manuscript did not require any funding. However, the base study was supported by the World Health Organization, Bangladesh (Agreement Reference: SEBAN140895) back in 2015. As a part of its mandate to strengthen national research capacity, WHO provided technical guidance in designing, implementing, analysing data, and writing the report. However, it does not have any influence on the results.

Consent to publish: All authors contributed to the manuscript and provided consent to publish.

Availability of data and materials: The dataset used for this manuscript is available with the corresponding author upon reasonable request.

Disclosure of Conflicts of Interest: None of the authors has any conflict of interest to disclose. The authors alone are responsible for their views expressed in this article, which do not necessarily represent the institutions' views, decisions, or policies with which they are affiliated.

Ethical Publication Statement: We confirm that we have read the Journal's position on issues involved in ethical publication and affirm that this report is consistent with those guidelines.

DRAFT

For peer review only

For peer review only - http://bmjopen.bmj.com/site/about/guidelines.xhtml

Tables

Table 1. Socioeconomic characteristics of patients of low back pain in Bangladesh, musculoskeletal disease survey 2015. (n=343)

Sociodemographic characteristics	Total (n=343)*	Man (n=126)	Woman (n=217)
	Number (percent)	Number (percent)	
Age in years, mean (standard deviation)	44.2 (13.8)	48.8 (13.0)	41.6 (13.6)
18-34	91 (26.5)	14 (11.1)	77 (35.5)
35-54	168 (49.0)	70 (55.6)	98 (45.2)
55-99	84 (24.5)	42 (33.3)	42 (19.4)
Occupation			
Homemaker	180 (52.5)	0 (0.0)	180 (82.9)
Laborer†	83 (24.2)	75 (59.5)	8 (3.7)
Business professional	22 (6.4)	22 (17.5)	0 (0.0)
Service holder	11 (3.2)	7 (5.6)	4 (1.8)
Others†	47 (13.7)	22 (17.5)	25 (11.5)
Education			
No formal education (0)	150 (43.7)	46 (36.5)	104 (47.9)
Any primary education (1-5)	71 (20.7)	33 (26.2)	38 (17.5)
Any secondary education (6-10)	97 (28.3)	38 (30.2)	59 (27.2)
Above secondary (≥11 years)	25 (7.3)	9 (7.1)	16 (7.4)
Married‡	334 (97.4)	123 (97.6)	211 (97.2)
Wealth index quartile§			
1st	103 (30.0)	38 (30.2)	65 (30.0)
2nd	92 (26.8)	34 (27.0)	58 (26.7)
3rd	71 (20.7)	26 (20.6)	45 (20.7)
4th	77 (22.5)	28 (22.2)	49 (22.6)
Rural residence	224 (65.3)	87 (69.0)	137 (63.1)
Duration of low back pain >3 months	253 (76.2)	103 (83.9)	149 (71.6)
Overweight (body mass index ≥25 Kg/m^2)	79 (23.0)	16 (12.7)	63 (29.0)
History of physical trauma	48 (14.0)	23 (18.3)	25 (11.5)
Current tobacco use	172 (50.1)	92 (73.0)	80 (36.9)
Concomitant musculoskeletal disorders‖	125 (36.4)	41 (32.5)	84 (38.7)
Hypertension¶	65 (19.0)	19 (15.1)	46 (21.2)
Diabetes mellitus**	18 (5.2)	2 (1.6)	16 (7.4)
Strenous physical activity††	51 (14.9)	49 (38.9)	2 (0.9)

* All values are number (percent) unless stated otherwise.

† Labourer includes: farmer, daily worker, rickshaw puller, garments worker, field worker and others. Other occupations: retired, weaver and housekeeper etc.

‡ Includes currently married, divorced, separated and widowed.

§ The wealth index was constructed using principal component analysis out of a list of 19 items (see Methods section for details);

‖ 125 respondents suffered from low back pain had either additional 1, 2 or 3 musculoskeletal disorders such as, knee osteoarthritis, soft tissue rheumatism, non-inflammatory musculoskeletal disorders, cervical spondylosis etc.

¶ Hypertension was defined as systolic blood pressure ≥140 or diastolic blood pressure ≥90 or medication for hypertension;

** Diabetes was defined as random capillary glucose level ≥11.1 or medication for diabetes;

†† Fourth quartile of the MET-minutes distribution of work-related physical activity. Commutation and leisure time physical activities were not considered because these were negligible contributors

DRAFT For peer review only

For peer review only - http://bmjopen.bmj.com/site/about/guidelines.xhtml

Table 2. Prevalence of low back pain by sociodemographic characteristics in Bangladesh, musculoskeletal disease survey 2015 (n=1843)

Sociodemographic characteristics	Total (n=1843)		Man (n=892)	Woman (n=951)
	Number	Percent (95% CI)	Percent (95% CI)	
Overall	343	18.6 (13.7–23.5)	14.1 (9.2–19.1)	22.8 (17.1–28.6)
Overall (age adjusted)[*]	-	19.4 (14.0–24.8)	14.0 (8.7–19.3)	27.2 (19.3–35.1)
Age in years				
18-34	711	12.8 (8.5–17.1)	6.5 (3.6–9.3)	15.6 (10.0–21.1)
35-54	774	21.7 (16.2–27.2)	16.6 (10.3–22.9)	27.8 (21.1–34.6)
55-99	358	23.5 (16.0–31.0)	16.6 (8.3–24.9)	40.0 (27.0–53.0)
Occupation				
Homemaker	749	24.0 (18.1–30.0)	0.0 (–)	24.0 (18.1–30.0)
Laborer[†]	472	17.6 (10.5–24.6)	17.2 (9.8–24.6)	17.6 (10.5–24.6)
Business professional	186	11.8 (6.4–17.3)	12.2 (6.6–17.7)	0.0 (–)
Service holder	135	8.1 (1.5–14.8)	6.5 (0.5–12.5)	14.8 (–)
Others[†]	301	15.6 (8.6–22.7)	13.3 (7.0–19.5)	18.5 (8.6–28.4)
Education				
No formal education (0)	564	26.6 (20.1–33.1)	17.2 (11.0–23.5)	35.0 (25.4–44.6)
Any primary education (1-5)	456	15.6 (9.4–21.8)	14.2 (6.9–21.4)	17.0 (8.8–25.3)
Any secondary education (6-10)	538	18.0 (12.1–24.0)	15.4 (7.6–23.2)	20.2 (13.8–26.6)
Above secondary (≥11 years)	285	8.8 (5.4–12.2)	6.2 (0.2–12.2)	11.5 (6.7–16.3)
Married[‡]	334	19.3 (14.2-24.3)	14.7 (9.5–19.9)	23.5 (17.5–29.5)
Wealth index quartile[§]				
1st	476	21.6 (14.5–28.8)	18.4 (11.7–25.2)	24.1 (14.5–33.6)
2nd	462	19.9 (11.4–28.4)	15.5 (6.4–24.5)	24.0 (14.5–33.5)
3rd	448	15.8 (10.8–20.9)	11.3 (6.0–16.5)	20.7 (13.3–28.2)
4th	457	16.8 (11.6–22.1)	11.9 (6.5–17.3)	22.1 (15.6–28.5)
Residence				
Urban	716	16.6 (11.6–21.6)	11.3 (7.4–15.2)	21.6 (13.4–29.7)
Rural	1127	19.9 (12.6–27.1)	15.9 (8.4–23.4)	19.9 (12.6–27.1)
Strenous physical activity[ǀ]	51	14.4 (7.9-20.9)	14.6 (7.4–21.4)	11.1 (–)
History of physical trauma	178	27.0 (19.5-24.4)	26 (15.9–36.4)	27.8 (15.4–40.2)
Current tobacco use	172	19.1 (14.1-23.9)	15 (9.9–20.1)	27.9 (20.0–35.8)

95% CI: 95% confidence interval
All values are number (percent) unless stated otherwise.
[*] Adjusted for WHO World Population 2000-2020.
[†] Labourer includes: farmer, daily worker, rickshaw puller, garments worker, field worker and others. Other occupations: retired, weaver and housekeeper
[‡] Includes currently married, divorced, separated and widowed.
[§] The wealth index was constructed using principal component analysis out of a list of 20 household assets (see Methods section for details);
[ǀ] Fourth quartile of the MET-minutes distribution of work-related physical activity. Commutation and leisure time physical activities were not considered because these were negligible contributors.
– 95% confidence interval not reported due number of respondents <25.

DRAFT
For peer review only

Table 3: Odds ratios of risk factors for low back pain in Bangladeshi adults, musculoskeletal disease survey 2015

Factors	Odds ratio (95% confidence interval)	
	Unadjusted	Adjusted
Age group, years	2.2 (1.7–2.9)**	2.5 (1.8–3.4)**
(35–99=1, 18–34=0)	1.0	1.0
Sex	1.9 (1.5–2.4)**	2.4 (1.8–3.2)**
(woman=1, man=0)	1.0	1.0
Labourer†	0.9 (0.7–1.1)	-
(yes=1, no=0)	1.0	-
No formal education	1.9 (1.5–2.5)**	1.5 (1.1–1.9)*
(yes=1, no=0)	1.0	1.0
Low wealth index	1.3 (1.0–1.6)	-
(yes=1, no=0)	1.0	-
Strenous physical activity‡	0.7 (0.5–0.9)*	1.0 (0.7–1.4)
(yes=1, no=0)	1.0	1.0
Overweight (body mass index ≥25 Kg/m^2)	1.3 (1.0–1.7)	-
(yes=1, no=0)	1.0	-
History of physical trauma	1.9 (1.3–2.7)**	1.9 (1.3–2.8)*
(yes=1, no=0)	1.0	1.0
Current tobacco user	1.1 (0.9–1.4)	-
(yes=1, no=0)	1.0	-
Hypertension	1.7 (1.3–2.4)**	1.4 (1.0–1.9)
(yes=1, no=0)	1.0	1.0
Diabetes mellitus	1.1 (0.6–1.9)	-
(yes=1, no=0)	1.0	-

LBP: low back pain; MSD: musculoskeletal disorder

* $P<0.05$

** $P<0.001$

† Labourer includes: farmer, daily worker, rickshaw puller, garments worker, field worker and others.

‡ Fourth quartile of the MET-minutes distribution of work-related physical activity. Commutation and leisure time physical activities were not considered because these were negligible contributors.

DRAFT For peer review only

For peer review only - http://bmjopen.bmj.com/site/about/guidelines.xhtml

Figures

Fig. 1 Flow chart on the selection of low back pain patients from the national survey on musculoskeletal conditions in Bangladesh (2015) adapted from Ahmad Zahid-Al-Quadir *et al* [21]

* Eight divisions from Sept 2015. Randomly 15 districts were selected out of 64.
** PPS indicates population proportion to size.
*** Out-migration, broken house, locked house, no tenant, out of home, refusal. Two recall visits were done if the selected house was locked and the person chosen was not available at home at the time of the interviewer's visit. In case of non-participation after the second recall visit, the targeted household/individual was declared non-respondents.

DRAFT
For peer review only

Fig. 2 Prevalence of LBP in different (Unadjusted overall prevalence (percent, 95% confidence interval) of low back pain and in) associated co-conditions (n=343)

*Diabetes was defined as random capillary glucose level ≥11.1 or medication for diabetes

†Overweight is defined as body mass index ≥25 Kg/m^2;

‡Hypertension was defined as systolic blood pressure ≥140 or diastolic blood pressure ≥90 or medication for hypertension;

§Respondents with multiple MSDs suffered from LBP, knee osteoarthritis, soft tissue rheumatism, non-inflammatory musculoskeletal disorders, cervical spondylosis etc.

For peer review only - http://bmjopen.bmj.com/site/about/guidelines.xhtml

445 **Fig. 3 Health assessment questionnaire – disability index (HAQ-DI) scores (A) and days lost from work (B) in low back pain (LBP) and no musculoskeletal disorder (MSD) groups.**

448

449 * Kruskal-Wallis H test

DRAFT
For peer review only

References

[1] Dionne CE, Dunn KM, Croft PR, et al. A consensus approach toward the standardization of back pain definitions for use in prevalence studies. Spine (Phila Pa 1976). 2008 Jan 1;33(1):95-103. doi: 10.1097/BRS.0b013e31815e7f94.

[2] Deyo RA, Tsui-Wu YJ. Descriptive epidemiology of low-back pain and its related medical care in the United States. Spine (Phila Pa 1976). 1987 Apr;12(3):264-8. doi: 10.1097/00007632-198704000-00013.

[3] Balagué F, Mannion AF, Pellisé F, et al. Non-specific low back pain. Lancet. 2012 Feb 4;379(9814):482-91. doi: 10.1016/S0140-6736(11)60610-7.

[4] Hoy D, Bain C, Williams G, et al. A systematic review of the global prevalence of low back pain. Arthritis Rheum. 2012 Jun;64(6):2028-37. doi: 10.1002/art.34347.

[5] Andersson GB. Epidemiological features of chronic low-back pain. Lancet. 1999 Aug 14;354(9178):581-5. doi: 10.1016/S0140-6736(99)01312-4.

[6] Fatoye F, Gebrye T, Odeyemi I. Real-world incidence and prevalence of low back pain using routinely collected data. Rheumatol Int. 2019 Apr;39(4):619-626. doi: 10.1007/s00296-019-04273-0.

[7] Hartvigsen J, Hancock MJ, Kongsted A, et al. Lancet Low Back Pain Series Working Group. What low back pain is and why we need to pay attention. Lancet. 2018 Jun 9;391(10137):2356-2367. doi: 10.1016/S0140-6736(18)30480-X.

[8] Parreira P, Maher CG, Steffens D, et al. Risk factors for low back pain and sciatica: an umbrella review. Spine J. 2018 Sep;18(9):1715-1721. doi: 10.1016/j.spinee.2018.05.018.

[9] Lötters F, Burdorf A. Prognostic factors for duration of sickness absence due to musculoskeletal disorders. The Clinical Journal of Pain. 2006 Feb;22(2):212-221. DOI: 10.1097/01.ajp.0000154047.30155.72.

[10] Guo HR, Tanaka S, Halperin WE, et al. Back pain prevalence in US industry and estimates of lost workdays. Am J Public Health. 1999 Jul;89(7):1029-35. doi: 10.2105/ajph.89.7.1029.

For peer review only - http://bmjopen.bmj.com/site/about/guidelines.xhtml

[11] Biglarian A, Seifi B, Bakhshi E, et al. Low back pain prevalence and associated factors in Iranian population: findings from the national health survey. Pain Res Treat. 2012;2012:653060. doi: 10.1155/2012/653060.

[12] Gupta G, Nandini N. Prevalence of low back pain in non working rural housewives of Kanpur, India. Int J Occup Med Environ Health. 2015;28(2):313-20. doi: 10.13075/ijomeh.1896.00299.

[13] Ahdhi GS, Subramanian R, Saya GK, et al. Prevalence of low back pain and its relation to quality of life and disability among women in rural areas of Puducherry, India. Indian J Pain [serial online] 2016 [cited 2021 Apr 18];30:111-5. Available from: https://www.indianjpain.org/text.asp?2016/30/2/111/186467.

[14] Bansal D, Asrar MM, Ghai B, et al. Prevalence and Impact of Low Back Pain in a Community-Based Population in Northern India. Pain Physician. 2020;23(4):E389-E98.

[15] Haq SA, Darmawan J, Islam MN, et al. Prevalence of rheumatic diseases and associated outcomes in rural and urban communities in Bangladesh: a COPCORD study. J Rheumatol. 2005 Feb;32(2):348-53. PMID: 15693098.

[16] Zahid-Al-Quadir, A., Zaman, M.M., Ahmed, S. et al. Prevalence of musculoskeletal conditions and related disabilities in Bangladeshi adults: a cross-sectional national survey. BMC Rheumatol 4, 69 (2020). https://doi.org/10.1186/s41927-020-00169-w

[17] Bangladesh Bureau of Statistics. Population and housing census 2011, preliminary result. Dhaka: Bangladesh Bureau of Statistics; 2001. Accessed 20 Apr 2020. http://bbs.portal.gov.bd.

[18] Islam, ME, Ahmed, S, Shahin, MA, et al. Cross-cultural adaptation and validation of revised WHO-ILAR-COPCORD core English questionnaire translated to Bangla. Int J Rheum Dis. 2021; 00: 1– 14. https://doi.org/10.1111/1756-185X.14176

[19] van Tulder M, Becker A, Bekkering T, et al. COST B13 Working Group on Guidelines for the Management of Acute Low Back Pain in Primary Care. Chapter 3. European guidelines for the management of acute nonspecific low back pain in primary care. Eur Spine J. 2006 Mar;15 Suppl 2(Suppl 2):S169-91. doi: 10.1007/s00586-006-1071-2.

For peer review only - http://bmjopen.bmj.com/site/about/guidelines.xhtml

[20] Zaman MM, Rahman MM, Rahman MR, et al. Prevalence of risk factors for non-communicable diseases in Bangladesh: Results from STEPS survey 2010. Indian J Public Health. 2016 Jan-Mar;60(1):17-25. doi: 10.4103/0019-557X.177290.

[21] Islam N, Baron Basak T, OudeVoshaar MA, et al. Cross-cultural adaptation and validation of a Bengali Health Assessment Questionnaire for use in rheumatoid arthritis patients. Int J Rheum Dis. 2013 Aug;16(4):413-7. doi: 10.1111/1756-185X.12032.

[22] Misra A. Ethnic-Specific Criteria for Classification of Body Mass Index: A Perspective for Asian Indians and American Diabetes Association Position Statement. Diabetes Technol Ther. 2015 Sep;17(9):667-71. doi: 10.1089/dia.2015.0007.

[23] Jaques H. Introducing the National Institute for Health and Clinical Excellence. Eur Heart J. 2012 Sep;33(17):2111-2. PMID: 23106069.

[24] Ahmad OB, Boschi-Pinto C, Lopez AD, et al. Age Standardization of Rates: A New WHO Standard. GPE Discussion Paper Series: No31 Geneva, Switzerland: World Health Organization, 2001.

[25] WMA Declaration of Helsinki - ethical principles for medical research involving human respondents. [Cited on: 2020 Aug 29] Available from: https://www.wma.net/policies-post/wma-declaration-of-helsinki-ethical-principlesfor-medical-research-involving-human-respondents/.

[26] Dutmer AL, Schiphorst Preuper HR, Soer R, et al. Personal and Societal Impact of Low Back Pain: The Groningen Spine Cohort. Spine (Phila Pa 1976). 2019 Dec 15;44(24):E1443-E1451. doi: 10.1097/BRS.0000000000003174.

[27] Al-Awadhi AM, Olusi SO, Al-Saeid K, et al. Incidence of musculoskeletal pain in adult Kuwaitis using the validated Arabic version of the WHO-ILAR COPCORD Core Questionnaire. Annals of Saudi Medicine. 2005 Nov-Dec;25(6):459-462. DOI: 10.5144/0256-4947.2005.459.

[28] Aldeen Sarakbi H, Alsaed O, Hammoudeh M, et al.. Epidemiology of musculoskeletal complaints and diseases in Qatar: A cross-sectional study. Qatar Med J. 2020 Nov 12;2020(2):29. doi: 10.5339/qmj.2020.29.

For peer review only - http://bmjopen.bmj.com/site/about/guidelines.xhtml

[29] Bansal D, Asrar MM, Ghai B, et al. Prevalence and Impact of Low Back Pain in a Community-Based Population in Northern India. Pain Physician. 2020 Jul;23(4):E389-E398. PMID: 32709185.

[30] Veerapen K, Wigley RD, Valkenburg H. Musculoskeletal pain in Malaysia: a COPCORD survey. J Rheumatol. 2007 Jan;34(1):207-13. PMID: 17216688.

[31] GBD 2017 Disease and Injury Incidence and Prevalence Collaborators. Global, regional, and national incidence, prevalence, and years lived with disability for 354 diseases and injuries for 195 countries and territories, 1990-2017: a systematic analysis for the Global Burden of Disease Study 2017. Lancet. 2018 Nov 10;392(10159):1789-1858. doi: 10.1016/S0140-6736(18)32279-7.

[32] Hoy D, Brooks P, Blyth F, et al. The Epidemiology of low back pain. Best Practice & Research Clinical Rheumatology. 2010 Dec;24(6):769–81. doi: 10.1016/j.berh.2010.10.002.

[33] Loney PL, Stratford PW. The prevalence of low back pain in adults: a methodological review of the literature. Phys Ther. 1999 Apr;79(4):384-96. PMID: 10201544.

[34] Manogharan S, Kongsted A, Ferreira ML, et al. Do older adults with chronic low back pain differ from younger adults in regards to baseline characteristics and prognosis? Eur J Pain (United Kingdom). 2017;21(5):866–73.

[35] Edwards J, Hayden J, Asbridge M, et al. The prevalence of low back pain in the emergency department: a descriptive study set in the Charles V. Keating Emergency and Trauma Centre, Halifax, Nova Scotia, Canada. BMC Musculoskelet Disord. 2018 Aug 23;19(1). doi: 10.1186/s12891-018-2237-x.

[36] Yue P, Liu F, Li L. Neck/shoulder pain and low back pain among school teachers in China, prevalence and risk factors. BMC Public Health. 2012 Sep 14;12(1). doi: 10.1186/1471-2458-12-789.

[37] Craft RM, Mogil JS, Aloisi AM. Sex differences in pain and analgesia: the role of gonadal hormones. European Journal of Pain. 2004 Oct;8(5):397–411. doi: 10.1016/j.ejpain.2004.01.003

For peer review only - http://bmjopen.bmj.com/site/about/guidelines.xhtml

[38] Berkley KJ. Sex differences in pain. Behav Brain Sci. 1997 Sep;20(3):371–80. doi: 10.1017/s0140525x97221485.

[39] Keogh E. Sex Differences in Pain. Reviews in Pain. 2008 Dec;2(2):4–7. doi: 10.1177/204946370800200203.

[40] Leboeuf-Yde C. Body Weight and Low Back Pain. Spine. 2000 Jan;25(2):226. doi: 10.1097/00007632-200001150-00015.

[41] Webb R, Brammah T, Lunt M, et al. Prevalence and Predictors of Intense, Chronic, and Disabling Neck and Back Pain in the UK General Population. Spine. 2003 Jun;28(11):1195–202. doi: 10.1097/01.BRS.0000067430.49169.01.

[42] Shmagel A, Foley R, Ibrahim H. Epidemiology of Chronic Low Back Pain in US Adults: Data From the 2009-2010 National Health and Nutrition Examination Survey. Arthritis Care & Research. 2016 Oct 26;68(11):1688–94. doi: 10.1002/acr.22890.

[43] Deyo RA, Tsui-wu YJ. Descriptive Epidemiology of Low-back Pain and Its Related Medical Care in the United States. Spine. 1987 Apr;12(3):264–8. doi: 10.1097/00007632-198704000-00013.

[44] Rubin DI. Epidemiology and Risk Factors for Spine Pain. Neurologic Clinics. 2007 May;25(2):353–71. doi: 10.1016/j.ncl.2007.01.004.

[45] Frymoyer JW, Pope MH, Costanza MC, et al. Epidemiologic Studies of Low-Back Pain. Spine. 1980 Sep;5(5):419–23. doi: 10.1097/00007632-198009000-00005.

[46] Coggon D, Ntani G, Palmer KT, et al. Drivers of international variation in prevalence of disabling low back pain: Findings from the Cultural and Psychosocial Influences on Disability study. European journal of pain. 2018 Jun 27;23(1):35–45. doi: 10.1002/ejp.1255.

[47] Harris IA, Young JM, Rae H, et al. Factors associated with back pain after physical injury: a survey of consecutive major trauma patients. Spine (Phila Pa 1976). 2007 Jun 15;32(14):1561-5. doi: 10.1097/BRS.0b013e318067dce8.

[48] Maher C, Underwood M, Buchbinder R. Non-specific low back pain. The Lancet. 2017 Feb;389(10070):736–47. doi: 10.1016/S0140-6736(16)30970-9.

For peer review only - http://bmjopen.bmj.com/site/about/guidelines.xhtml

[49] Bae Y-H, Shin J-S, Lee J, et al. Association between Hypertension and the Prevalence of Low Back Pain and Osteoarthritis in Koreans: A Cross-Sectional Study. Fuchs FD, editor. PLoS ONE. 2015 Sep 22;10(9):e0138790. doi: 10.1371/journal.pone.0138790.

DRAFT
For peer review only

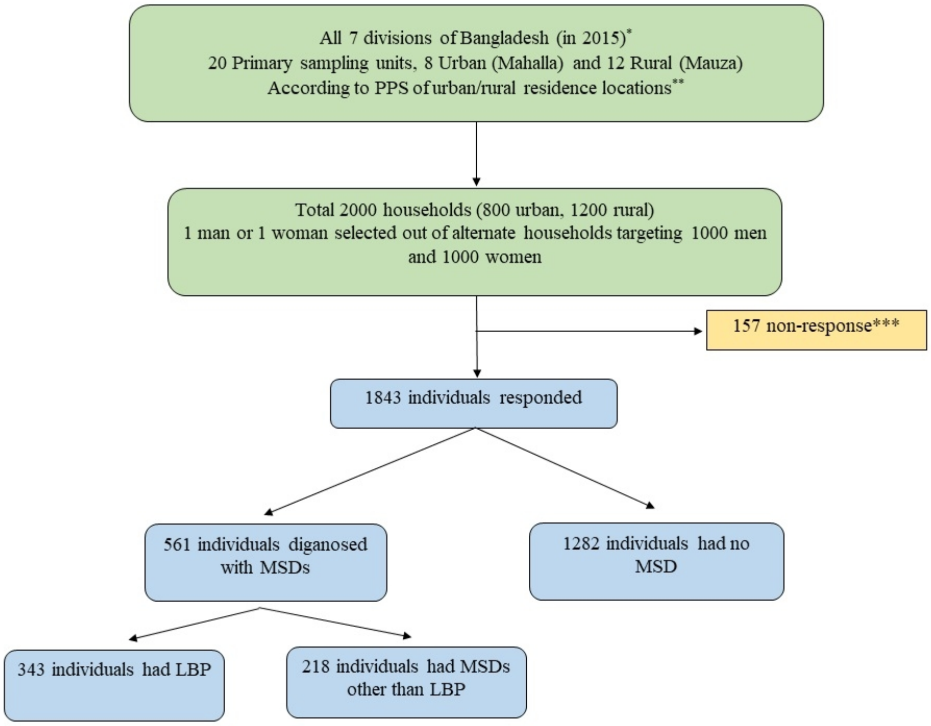

254x190mm (96 x 96 DPI)

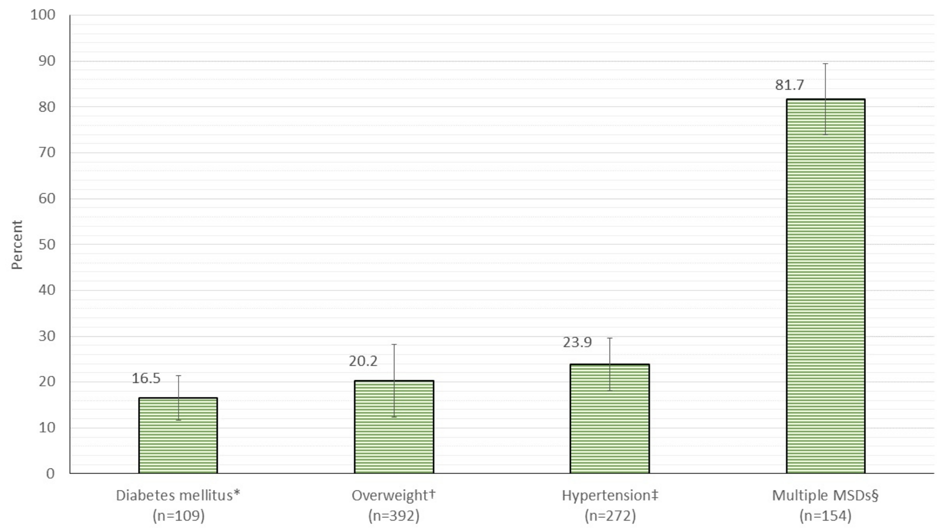

338x190mm (96 x 96 DPI)

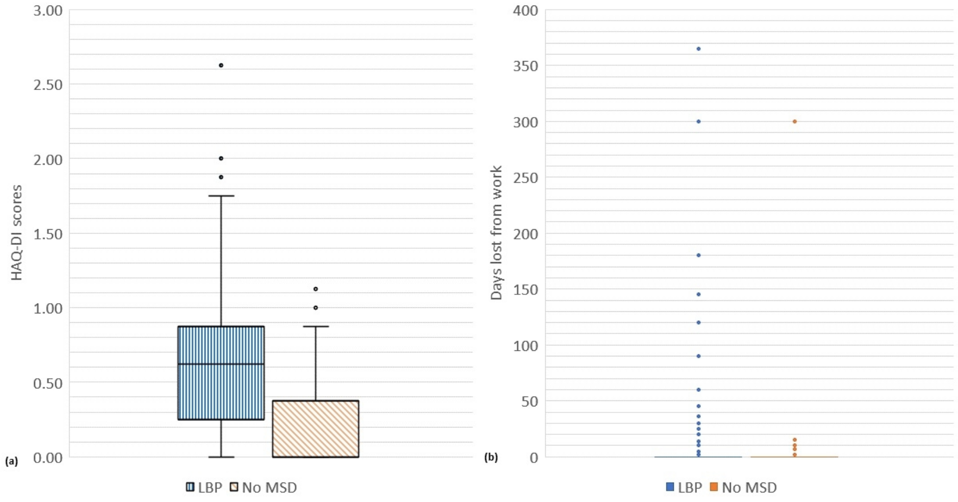

338x190mm (96 x 96 DPI)

BMJ Open

Low Back Pain in the Bangladeshi Adult Population: A Cross-sectional National Survey

Journal:	*BMJ Open*
Manuscript ID	bmjopen-2021-059192.R1
Article Type:	Original research
Date Submitted by the Author:	06-Jul-2022
Complete List of Authors:	Momen Majumder, Muhammad Shoaib; Bangabandhu Sheikh Mujib Medical University, Rheumatology<br>Hakim, Ferdous; World Health Organization Bangladesh<br>Bandhan, Iftekhar ; Bangabandhu Sheikh Mujib Medical University, Rheumatology<br>Razzaque, Mohammad Abdur; Chittagong Medical College, Medicine<br>Zahid-Al-Quadir, Ahmad ; Sylhet MAG Osmani Medical College, Medicine<br>Ahmed, Shamim; Bangabandhu Sheikh Mujib Medical University<br>Choudhury, Minhaj; Bangabandhu Sheikh Mujib Medical University, Rheumatology<br>Haq, Syed; Bangabandhu Sheikh Mujib Medical University, Rheumatology<br>Zaman, MM; WHO Bangladesh
**Primary Subject Heading**:	Epidemiology
Secondary Subject Heading:	Rheumatology
Keywords:	EPIDEMIOLOGY, RHEUMATOLOGY, PUBLIC HEALTH, PREVENTIVE MEDICINE

SCHOLARONE™
Manuscripts

I, the Submitting Author has the right to grant and does grant on behalf of all authors of the Work (as defined in the below author licence), an exclusive licence and/or a non-exclusive licence for contributions from authors who are: i) UK Crown employees; ii) where BMJ has agreed a CC-BY licence shall apply, and/or iii) in accordance with the terms applicable for US Federal Government officers or employees acting as part of their official duties; on a worldwide, perpetual, irrevocable, royalty-free basis to BMJ Publishing Group Ltd ("BMJ") its licensees and where the relevant Journal is co-owned by BMJ to the co-owners of the Journal, to publish the Work in this journal and any other BMJ products and to exploit all rights, as set out in our licence.

The Submitting Author accepts and understands that any supply made under these terms is made by BMJ to the Submitting Author unless you are acting as an employee on behalf of your employer or a postgraduate student of an affiliated institution which is paying any applicable article publishing charge ("APC") for Open Access articles. Where the Submitting Author wishes to make the Work available on an Open Access basis (and intends to pay the relevant APC), the terms of reuse of such Open Access shall be governed by a Creative Commons licence – details of these licences and which Creative Commons licence will apply to this Work are set out in our licence referred to above.

Other than as permitted in any relevant BMJ Author's Self Archiving Policies, I confirm this Work has not been accepted for publication elsewhere, is not being considered for publication elsewhere and does not duplicate material already published. I confirm all authors consent to publication of this Work and authorise the granting of this licence.

For peer review only

Version: 2.4 date: 20 June 2022

Low Back Pain in the Bangladeshi Adult Population: A Cross-sectional National Survey

Short Running Head: *Low Back Pain in Bangladesh*

Authors: Muhammad Shoaib Momen Majumder, Ferdous Hakim, Iftekhar Hussain Bandhan, Mohammad Abdur Razzaque, Ahmad Zahid-Al-Quadir, Shamim Ahmed, Minhaj Rahim Choudhury, Syed Atiqul Haq, Mohammad Mostafa Zaman

Author names, highest academic degrees, appointments, Email and ORCID:

Muhammad Shoaib Momen Majumder, FCPS (Medicine), MD (Rheumatology); Department of Rheumatology, Bangabandhu Sheikh Mujib Medical University (Ringgold ID 74464), Bangladesh, Email: momen.shoaib@gmail.com, ORCID ID: https://orcid.org/0000-0002-7901-0847;

Ferdous Hakim, MPH (Epidemiology), Research and Publication, World Health Organization Bangladesh (Ringgold ID 215134), Email: hakimf@who.int, ORCID: https://orcid.org/0000-0003-2376-3978;

Iftekhar Hussain Bandhan, MD (Rheumatology), Department of Rheumatology, Bangabandhu Sheikh Mujib Medical University (Ringgold ID 74464), Bangladesh, Email: ibandhan@gmail.com, ORCID ID: https://orcid.org/0000-0002-3800-6155;

Mohammad Abdur Razzaque, MD (Rheumatology), Register, Department of Medicine, Chittagong Medical College (Ringgold ID 467859), Bangladesh, Email: rhrazzaq@gmail.com, ORCID ID: https://orcid.org/0000-0002-2395-542X;

Ahmad Zahid-Al-Quadir, MD (Rheumatology), Department of Medicine, Sylhet MAG Osmani Medical College (Ringgold ID 469196), Email: drzahidssmc@gmail.com, https://orcid.org/0000-0003-4178-6487;

Shamim Ahmed, FCPS (Medicine), Associate Professor of Rheumatology, Bangabandhu Sheikh Mujib Medical University (Ringgold ID 74464), Dhaka, Bangladesh, Email: dr_shamim_ahmed@yahoo.com;

Minhaj Rahim Choudhury, MD, FCPS (Medicine) Professor of Rheumatology, Bangabandhu Sheikh Mujib Medical University (Ringgold ID 74464), Dhaka, Bangladesh, Email: drminhaj11@gmail.com, ORCID ID: https://orcid.org/0000-0002-8695-5240;

Syed Atiqul Haq, FCPS (Medicine), MD, FRCP, Professor of Rheumatology, Bangabandhu Sheikh Mujib Medical University (Ringgold ID 74464), Dhaka, Bangladesh, Email: haqsyedatiqul@gmail.com, ORCID ID: https://orcid.org/0000-0003-4154-7283

Mohammad Mostafa Zaman, PhD (Epidemiology), Advisor, Research and Publication, World Health Organization Bangladesh (Ringgold ID 215134), Email: zamanm@who.int, ORCID ID: https://orcid.org/0000-0002-1736-1342;

Counts: Number of words (abstract): 264, number of words (body): 3580, number of references: 61, number of tables: 04, number of figures: 02

Name, address, email of the author responsible for correspondence:

For peer review only - http://bmjopen.bmj.com/site/about/guidelines.xhtml

Version: 2.4 date: 20 June 2022

Muhammad Shoaib Momen Majumder, FCPS (Medicine), MD (Rheumatology); Department of Rheumatology, Bangabandhu Sheikh Mujib Medical University (Ringgold ID 74464), Bangladesh, Email: momen.shoaib@gmail.com, ORCID ID: https://orcid.org/0000-0002-7901-0847

ABSTRACT

Objective:

Low back pain (LBP) is a common musculoskeletal disorder. This study aims to determine the residence- and sex-specific prevalence and the risk factors of LBP in Bangladesh.

Methods:

The study subjects (ages ≥18 years) were identified from 20 primary sampling units of the national census following a cross-sectional multi-stage stratified sampling design. We considered the mechanical type of LBP for this study. A Bangla version of the modified Community Oriented Program for Control of Rheumatic Disorders questionnaire was used. A team of trained field workers, rheumatology residents and rheumatologists collected the data. Analysis was done using weighted data.

Results:

Two thousand subjects were approached, but 1843 could be screened. Among them, 561 had musculoskeletal disorders, and 343 were diagnosed with LBP. The weighted prevalence of LBP was 18.5% (95% confidence interval, CI: 11.8–25.2) and age-standardized prevalence of LBP was 19.4% (95% confidence interval, CI: 14.0–24.8), which was higher in women (27.2%, 19.3-35.1) than men (14.0%, 8.7-19.3). The prevalence persistently increased from the age group 18-34 years (10.5%, 5.7–15.4) to ≥55 years (27.8%, 16.1–39.5). People with no education had the highest prevalence (31.3%, 22.3–40.4). The prevalence did not differ between urban and rural residential locations. Four factors were significantly associated with LBP: age (adjusted odds ratio (2.4, 95% CI: 1.7–3.4), female sex (2.2, 1.5–3.3), absence of formal education (2.3, 1.6–3.3), and hypertension (1.7, 1.1–2.6).

Conclusion:

For peer review only - http://bmjopen.bmj.com/site/about/guidelines.xhtml

LBP is a common problem in Bangladeshi adults. The risk factors are age, female sex, no formal education, and hypertension. These should be addressed adequately to prevent and treat LBP.

Keywords: Low back pain, risk factors, Bangladesh, prevalence, cross-sectional survey

Strengths and Limitations

- We report the weighted prevalence of low back pain by sociodemographic characteristics, co-morbidities, disability and work loss and identified risk factors for low back pain patients for the first time in Bangladesh.
- All the diagnoses were made by rheumatology residents and expert rheumatologists in the field.
- Some diagnoses of evolving rheumatological conditions might lack validity because of the lack of quality laboratory facilities in the field.
- The sample size calculation is based on the combined prevalence of musculoskeletal disorders that warrant cautious interpretation of the results because of inadequate sample size, especially when split into reporting domains.
- The recall period for determining work loss was 12 months which might induce bias.

For peer review only - http://bmjopen.bmj.com/site/about/guidelines.xhtml

INTRODUCTION

Low back pain (LBP) is one of the most frequent medical problems globally[1]. It is defined as pain, stiffness, or muscle tension localised below the costal margin and above the inferior gluteal folds[2]. Up to 84% of adults suffer from LBP at some point in life[3]. The prevalence of chronic LBP is about 23%[4]. Around 11–12% of the population become disabled due to LBP[1]. It causes substantial personal, social and financial burdens globally[1]. In the USA, LBP is the second most frequent cause for a physician consultation[5]. LBP is ranked globally as the topmost cause of disability as it affects mostly working-age people[6]. It accounted for 60.1 million disability-adjusted life-years in 2015[7]. There was a significant increase in LBP by 54% since 1990, and the highest escalation took place in the low and middle-income countries (LMICs)[7]. Disability from LBP is a primary concern for the LMICs, including Bangladesh, where manual labour–rickshaw pulling, day labourers, housemaids, work exposure to the lifting of heavy weight during their day-to-day activities etc.–is common*7*. The scope for job switching is restricted in resource constraint countries.

LBP has multi-sectorial health outcomes like a lower quality of life, poorer self-reported health, depression and more workspace absenteeism[8]. As a result, LBP has become an important cause of sick leave and early retirement among the working population[9]. In the USA, approximately 149 million workdays are lost due to LBP, leading to an estimated loss of 100–200 billion US dollars per year[10]. Non-specific LBP is the commonest of all causes of LBP[4]. Non-specific LBP is defined as LBP not particularly attributable to specific aetiology like malignancy, infection, fracture, inflammatory condition, radiculopathy or cauda equina syndrome[4].

Albeit high in most studies, there is a difference in LBP prevalence in various epidemiological studies. The estimated lifetime prevalence was 84.1% in a Canadian study[11], 70% in Denmark[12], and 59% in the UK[13]. In Iran, the prevalence of LBP was 29.3%[14]. The estimated prevalence of LBP in India ranged between 42 and 83%[15 16]. A recent cross-sectional, community-based, epidemiological study conducted in Northern India yielded an estimated lifetime prevalence of 47% in man 57% in women[17]. A Community Oriented Program for Control of Rheumatic Disorders (COPCORD) survey in Bangladesh published in 2005 showed 6.6%, 9.9%, and 9.2% prevalence of LBP in the rural, urban slum, and

For peer review only - http://bmjopen.bmj.com/site/about/guidelines.xhtml

affluent urban areas, respectively[18]. A cross-sectional national study in Bangladesh in 2015 showed LBP was the top-ranking musculoskeletal disorder (MSD) with a prevalence of 18.6%[19]. We have further analysed the data from the 2015 study and reported the population-weighted prevalence according to sociodemographic factors, co-morbid conditions, disability and work loss due to LBP, and identified the risk factors of LBP.

METHODS

A detailed description of the methodology is beyond the scope of this article and is described elsewhere[19]. Adults aged 18 years or more comprised the study population through a household-level multi-stage stratified cross-sectional survey. The sampling frame was based on the 2001 Bangladesh Census[20]. Based on a point prevalence of MSD and with a design effect of 1.5 and 85% response rate for four reporting domains (man-woman, urban-rural), the calculated sample size was 1,978, which was rounded to 2,000. It was stratified into seven divisions of rural (Mauza) and urban (Mahalla) areas. Twenty (8 urban and 12 rural) primary sampling units (PSUs) were selected. The first 100 households were consecutively included from each PSU, where even numbers were assigned as man and odd numbers as woman households. In each household, a single respondent was identified from a list of eligible household members with the help of a Kish table. Data were collected in November and December of 2015. (**Figure 1**)

A detailed manual was prepared before the training for this survey and was used by all field staff. All investigators and the WHO technical team coordinated and conducted the training. The modified COPCORD questionnaire was the survey tool[21]. The English version of the first part of the questionnaire was translated to Bangla, then adapted according to the guideline of Beaton et al.[22], validated by Chassany's Method[23], and administered by the interviewers. Data were collected for six days from each PSU. There were two recall visits to ensure participation. The research physician interviewed the suspected respondents for MSDs. A subject was considered a positive respondent if he/she reported pain in muscles, bones, joints, or any part of the body (musculoskeletal system) during the preceding seven days. Subjects who were taking pain medications like non-steroidal anti-inflammatory drugs (NSAID) or steroids were considered positive respondents even if they did not report pain on those seven days. All positive respondents were interviewed and examined by the research

For peer review only - http://bmjopen.bmj.com/site/about/guidelines.xhtml

physicians. Internationally accepted criteria were used for the diagnosis of the diseases. For the conditions without any internationally accepted criteria, relevant investigations and clinical judgment of the research physician were used. A rheumatologist checked and verified the final diagnoses during their visit to respective PSUs.

LBP group of disorders were operationally defined as mechanical type back pain that included non-specific LBP and lumbar spondylosis. Considering the limitation of differentiating investigation in the field, we did not classify LBP beyond this. LBP duration was classified into three groups: acute: up to 6 weeks; subacute: 6-12 weeks; and chronic, which persists beyond 12 weeks[24]. Respondents with pain in the muscles, bones, joints, or any part of the body (musculoskeletal symptom) during the preceding seven days or on pain medication with no pain were considered positive respondents. The research physicians interviewed and thoroughly examined all 'positive' respondents. Data on physical activity were calculated into metabolic equivalent tasks (MET)-minutes per week using the STEPwise Surveillance of noncommunicable disease risk factors (STEPS) protocol and divided into quartiles[25]. The 4th quartile was labelled as strenuous physical activity.

The study participants were divided into three subgroups as per age in years: 18-34, 35-54 and 55-99. We considered ownership of household asset items (electricity, television, refrigerator, etc.) for constructing wealth index. In addition, the type of main material used for the roof of the main house (cement, tin and katcha such as bamboo/thatched/straw/gunny etc.) was also included in the model. A principal component analysis was used to create standardised factor scores for each of the items. The total scores for the respondents were calculated and categorised into quartiles for description from one (lowest household wealth) to four (highest household wealth)[19].

A validated Bangla version of the Bangla Health Assessment Questionnaire - Disability Index (HAQ-DI) was used for the disability score. For determining work loss, the recall period was 12 months[26]. Random capillary blood glucose was measured by using glucometer (Accu-Check Germany). Using height (meters) and weight (kilograms) measurements, we calculated BMI [weight (kg)/height (meter)^2]. Waist circumference was measured by placing a measuring tape horizontally above the iliac crest. Diabetes was defined as random blood

For peer review only - http://bmjopen.bmj.com/site/about/guidelines.xhtml

glucose ≥11.1 mmol/l or the use of anti-diabetic medications. Obesity was defined as a body mass index of ≥25 kg/meter squared [27].

Statistical analysis:

The survey data were entered and cleaned using Microsoft Excel. We have weighted[28] the data to reflect the population frame of Bangladesh for the year 2015. Base weight–for the sampled population–was calculated using the probability of selection of respondents among the eligible number of members of the household in a cluster defined by divisions (7), age groups (3) and sexes (2). The base weight was adjusted with non-response weights separately for men and women by three age groups. The final weight was generated after calibration to frame population (2015) in domains by division, sex and age groups. Analysis was done using the final weights.

Statistical analysis was done using Epi Info Version 7.1.5.2 and SPSS Version 20.0. Continuous variables were categorised before analysis as appropriate. We estimated the prevalence of LBP with 95% confidence intervals. The prevalence was segregated by residence (urban/ rural) and sex (man/ woman). Nonparametric test (Kruskal-Wallis H test) was used to analyse data that were not normally distributed. The confidence intervals were suppressed whenever we encountered an unweighted respondent size of less than 25. Age-standardization of prevalence estimates was made for global comparison using the WHO World Population 2000-2025[29]. Factors were checked for association with LBP by comparing LBP with no MSD through 2x2 tables. Univariate logistic regression analysis was done to obtain unadjusted odds ratios. All statistically significant relationships (P<0.05) were entered into a model for logistic regression analysis. The adjusted odds ratios and their 95% confidence limits were calculated to identify the strength of association of LBP factors. A detailed description of categorisation and analysis of other variables was described elsewhere[19].

Ethics Approval and Consent to Participate:

Ethical guidelines, as outlined by the Declaration of Helsinki, were followed throughout the study[30]. Ethical clearance was obtained from the Institutional Review Board of Bangabandhu

For peer review only - http://bmjopen.bmj.com/site/about/guidelines.xhtml

Sheikh Mujib Medical University (ID 1100). Informed written consent was obtained from the respondents in Bangla per Institutional Review Board's guidelines.

Patient and Public Involvement:

Patients or the public were not involved in this study's design, conduct, reporting, or dissemination plans.

RESULTS

Characteristics of respondents:

In this nationally representative study, 2,000 adults 18 years or older were approached, and 1,843 (92.2%) agreed to participate[19]. The mean age of the participants was 40.5 (standard deviation 14.7) years, and 51.6% were women. A total of 561 (30.4%) had some type of MSDs. LBP was the most common among MSDs (18.6%, unadjusted), followed by knee osteoarthritis (7.3%) and soft-tissue rheumatism (5.2%). Among the inflammatory rheumatic diseases, the common conditions were rheumatoid arthritis (1.6%) and spondyloarthritis (1.3%).

Table 1 shows the changes brought in by the weighting procedure on the unweighted sample. The weighted percentages are more in line with the projected Population Frame[20] from which the study sample was drawn.

Characteristics of respondents with LBP:

Prevalence:

We report here (**Table 2**) weighted prevalence of LBP was 18.5% (95% CI: 11.8–25.2). However, the age-standardised prevalence of LBP is 19.4% (95% CI: 14.0–24.8), which is significantly higher in women (27.2%, 19.3-35.1) than in men (14.0%, 8.7–19.3). There has been a persistent increase in prevalence from 10.5% (95% CI 5.7–15.4) in the 18–34 years age group to 27.8% (95% CI 16.1–39.5) in the 55–99 years age group. This trend was more prominent in women. The prevalence did not vary significantly among occupational groups. People with no formal education had a significantly highest prevalence of LBP (31.3%, 22.3–40.4) compared to other educational groups. Although the highest prevalence (23.5% (13.9–33.0)) was observed in the 1st quartile of the wealth index, it did not vary significantly. LBP

For peer review only - http://bmjopen.bmj.com/site/about/guidelines.xhtml

was not significantly associated with strenuous physical activity in our sample. We checked LBP prevalence in urban (14.6%, 9.9–19.2) and rural (20.2%, 11.0.6–29.3) categories, but it did not differ significantly. Among the co-morbidities, the prevalence of LBP was higher among patients with hypertension (26.7%, 15.0–38.4) and obesity (20.6%, 13.0–28.3). The highest prevalence of LBP (87.3%, 80.2–94.4) was seen in respondents who had multiple (two or more) MSDs such as LBP, knee osteoarthritis, soft tissue rheumatism, non-inflammatory MSDs, cervical spondylosis etc. **(Figure 2)**

Background characteristics:

Among the LBP respondents (n=343), 63.3% were women, and 65.3% were from rural areas. The mean age in years (95% confidence interval) was 45.3 (43.0–47.7) overall, and 48.3 (45.8–50.9) in men and 44.0 (41.0–47.0) in women. The study participants were divided into three subgroups per age, and the highest LBP was observed in the 35–54 age group. More than half (%, 95% confidence interval: 57.4%, 48.2–66.6) were homemakers (all women), while the rest constituted other occupations like a labourer, business professional, service holder and others. Almost half of the participants with LBP had no formal education (53.2%, 41.6–64.9). Overall, according to the wealth index, 33.2% (22.6–43.9) of respondents belonged to the 1st quartile (lowest socioeconomic status). About three-fourths of the respondents (77%, 55.9–98.0) had a rural residence. **(Table 3)**

Disability and work loss:

The distribution of Bengali HAQ-DI scores was not normally distributed among the LBP patients. The scores ranged from 0–2.6. The LBP group's median (interquartile range) was 0.6 (0.3–0.9). The difference in Bangla HAQ-DI score between LBP (n=343) and no MSD (n=60) is statistically significant (P<0.0001) by the Kruskal-Wallis H test, indicating that LBP is associated with a higher disability. The LBP group's days lost from work had a highly skewed distribution and ranged from 0–365 days. However, the difference of days lost from work (n=1,625) between LBP (n=343) and no MSD (n=1,282) is statistically significant (P<0.0001) by the Kruskal-Wallis H test, indicating that LBP is associated with more days lost.

Risk factors:

DRAFT
For peer review only

For peer review only - http://bmjopen.bmj.com/site/about/guidelines.xhtml

Univariate logistic regression analysis did not show any significant association of LBP with occupation, strenuous physical activity, wealth indices, tobacco use, obesity, and diabetes (**Table 4**). A significant association was observed for the age group 35–99 years, female sex, lack of education, history of physical trauma, and hypertension according to the unadjusted odds ratio and their 95% confidence intervals ($P<0.05$). These significant associations ($P<0.01$) persisted in the multiple logistic regression analysis having age, sex, education and hypertension into the model simultaneously.

DISCUSSION

LBP is a common medical problem with very high personal and societal impact, leading to poor quality of life and workability[31]. In this survey, we report that one in five adults in Bangladesh suffers from LBP. The prevalence in Bangladesh is similar to Kuwait (22.7%)[32] and Qatar (23.3%)[33] but lower than northern India (32%)[34] and Iran (29.3%)[14]. Malaysia (11.6%)[35] has a lower prevalence of LBP than we report here. In some previous studies in Bangladesh, the prevalence was found at 25.6% among medical students[36], 36.6% among bank employees[37], and 60.8% among physiotherapists[38]. Lack of maintenance of correct posture during sitting and scarcity of knowledge, understanding, or application of ergonomics are responsible for the high prevalence rate among these groups[36 38].

A systematic review of 165 studies from 54 countries revealed the global prevalence of LBP of 12-33%[1]. According to the systematic analysis of the Global Burden of Disease Study 2017, LBP was the leading cause of YLDs (years lived with disability)[39]. In our study, the rural people had a higher prevalence of LBP than the urban people. This difference–though not statistically significant–was probably due to lower doctor concentration in the rural areas, financial limitations, and less education status. However, regional variation was observed in other studies[14].

Age was a risk factor for LBP in this study. LBP prevalence persistently increased with age but was not statistically significant. However, an analysis reported that the risk and prevalence of low back pain increased with age[5]. A systematic review of the global prevalence of LBP revealed the association of age was highest in the 40-49 age group[1 40]. The overall prevalence rises with age 65, which gradually reduces after that[41]. Some possible

For peer review only - http://bmjopen.bmj.com/site/about/guidelines.xhtml

explanations are LBP characteristics in older adults that differ from the middle-aged population (less intense back pain, more leg pain, and more depression)[42]. Our study showed a higher prevalence of LBP among women than men, consistent with some other analyses[1 41]. This could be due to more household or domestic activities among women than men. This finding agrees with the results from the national health survey on the Iranian population[14]. Another Indian study found no significant difference in age and sex scores in their study[43]. Higher women's prevalence can be partially explained that they have a lower pain threshold than men[44]. The sex differences may be implied by gonadal steroid hormones like estradiol and testosterone that modulate sensitivity to pain and analgesia[45]. Women always experienced a higher frequency of visceral pain (e.g., during menstruation, pregnancy) than men[46]. It seems that in painful conditions, women exhibit a greater prevalence than men as women report more pain[47].

Leboeuf-Yde considered body weight as a possible weak indicator for LBP in his systematic literature review due to lack of evidence[48]. The results obtained in our study did not demonstrate a statistically significant association between LBP and higher BMI. Some other studies found obesity or high BMI associated with an increased risk of LBP development and severity[14 49]. However, a cross-sectional study including nine countries found BMI ≥25kg/m^2 as a risk factor for LBP in five countries (Finland, Poland, Russia, South Africa and Spain). In contrast, it was not associated with LBP in the remaining four countries (China, Ghana, India and Mexico)[50].

Our research found that the absence of formal education is significantly associated with LBP. A cross-sectional study in the United States found that LBP is more common in people who have had less than high school education[51]. Other studies in the USA, UK, and Iran found lower educational status had an increased association with LBP and found higher education inversely associated with LBP[14 52]. Several proposed mechanisms may explain the relationship between low academic status and back pain. The amount of formal education contributes to the types of jobs that an individual may involve in, and subsequently, the types of jobs influence LBP[53]. Moreover, health education regarding posture management, lifestyle changes, physical exercises, and stress management was poorly reached among people without formal education. We didn't find any significant association of LBP with occupation.

For peer review only - http://bmjopen.bmj.com/site/about/guidelines.xhtml

However, in our opinion, the larger number of homemakers affected with LBP might be linked with their nature of heavy work, such as squatting, bending, lifting heavy objects, prolonged standing etc., in the household. In a US-based study, LBP was significantly related to occupational factors such as truck driving, lifting, carrying, pulling, pushing, twisting, and non-driving vibrational exposure[54]. In some European countries, workers involved in heavy weightlifting (≥25 kg) suffered more from LBP[55].

Studies reported associations between LBP and lower social class[14 51], but we did not find any significant association of LBP with economic status. This finding is coherent with a Danish study that did not find any possible relationship between socioeconomic status and LBP[56]. In our analysis, trauma tended to be associated (unadjusted OR) with overall LBP, but the association was lost after adjustment. Trauma is not supposed to lead to chronic LBP, and the persistence of back pain was more associated with psychological factors like stress, low education status, etc., than the trauma itself[57]. We found a positive relationship between hypertension and LBP. LBP may cause inactivity and lack of exercise resulting in weight gain, subsequently creating or exaggerating co-morbid conditions like hypertension and diabetes mellitus. The Hong Kong Disc Degeneration-Cardiovascular Cohort showed that HTN increases the possibility of moderate or severe disc degeneration, which is highly associated with LBP[58]. Another long-term Finish study revealed that both SBP and DBP were positively associated with LBP suggesting atherosclerosis of lumbar vessels as a possible mechanism of development of LBP[59]. In a Korean survey, the lifetime prevalence of LBP was 34.4% among the hypertensive respondents, but the adjusted OR of LBP prevalence was significantly lower than the normotensive subjects (fully adjusted 0.79, 95% CI 0.70-0.90)[60]. A Bangladeshi study conducted among the doctors working in a tertiary care hospital found that HTN was the most common co-morbid condition among the LBP sufferers[61]. There was no association between diabetes and LBP in this study.

Strengths and Limitations

This is the first nationally representative survey report on LBP in Bangladesh and probably, among all south Asian nations. Although we have weighted the data for national representation, the sample size calculation for the original study was based on the point prevalence of MSD[19]. We now know that the prevalence of LBP was 18.5%, and the

DRAFT
For peer review only

prevalence of MSD was 30.4%. A larger sample size maintaining adequate power was needed for the generalizability of the study results. Therefore, a cautious interpretation is necessary because of the inadequate sample size, especially when split into reporting domains. We have operationally defined the recall period for reporting work loss days as 12 months which might induce bias. Trained rheumatology residents diagnosed the patients, which the experienced rheumatologists verified in the field. Some diagnoses of evolving rheumatological conditions might lack sufficient validity because of inadequate laboratory facilities in the field.

CONCLUSION

This nationally representative study reports the population-weighted prevalence of LBP by sociodemographic background, co-morbidities and risk factors in Bangladesh. One in five adults suffers from LBP. Education and hypertension are modifiable risk factors that warrant intervention. An increase in the level of education, care for the middle and older population, and good control of hypertension may reduce the LBP burden. Special attention is needed to prevent LBP in women. Further study with a larger sample size addressing these neglected issues may have more clarifications to decrease the burden of LBP.

For peer review only - http://bmjopen.bmj.com/site/about/guidelines.xhtml

Abbreviations and acronyms:

BMI	Body mass index
BSMMU	Bangabandhu Sheikh Mujib Medical University
CI	Confidence interval
COPCORD	Community oriented program from control of rheumatic diseases
HAQ-DI	Health Assessment Questionnaire - Disability Index
LBP	Low back pain
MSK	Musculoskeletal
MSD	Musculoskeletal disorder
OR	Odds ratio
PPS	Population proportion to size
PSU	Primary sampling unit
WHO	World health organisation

Acknowledgement: We gratefully acknowledge the field team, divisional coordinators, civil surgeons, upazila health and family planning officers and health assistants for their support.

Author contribution:

Muhammad Shoaib Momen Majumder (MSMM): Conceptualised the area of work, and prepared the first draft of the manuscript and revised the subsequent drafts.

Ferdous Hakim (FH): analysed the data, guided preparation of graphs and tables, interpreted the results, revised the draft manuscript, and coordinated the writing exercise.

Iftekhar Hussain Bandhan (IHB): prepared graphs and tables, revised the draft manuscript.

Mohammad Abdur Razzaque (MAR): reviewed the manuscript and with special reference to the literature review.

Ahmed Zahid-Al-Quadir and Shamim Ahmed: Prepared the training manual, trained the field team, executed the field operation in coordination with the divisional investigators;

Minhaj R Choudhury, Syed Atiqul Haq and Mohammad Mostafa Zaman: Designed the study, guided the analysis and manuscript writing, critically reviewed the results and manuscript drafts, ensured integrity of data.

All authors: Approved publication.

For peer review only - http://bmjopen.bmj.com/site/about/guidelines.xhtml

Funding: Preparing this manuscript did not require any funding. However, the base study was supported by the World Health Organization, Bangladesh (Agreement Reference: SEBAN140895) back in 2015. As a part of its mandate to strengthen national research capacity, WHO provided technical guidance in designing, implementing, analysing data, and writing the report. However, it does not have any influence on the results.

Consent to publish: All authors contributed to the manuscript and provided consent to publish.

Availability of data and materials: The dataset used for this manuscript is available with the corresponding author upon reasonable request.

Disclosure of Conflicts of Interest: None of the authors has any conflict of interest to disclose. The authors alone are responsible for their views expressed in this article, which do not necessarily represent the institutions' views, decisions, or policies with which they are affiliated.

Ethical Publication Statement: We confirm that we have read the Journal's position on issues involved in ethical publication and affirm that this report is consistent with those guidelines.

For peer review only - http://bmjopen.bmj.com/site/about/guidelines.xhtml

442 **Tables**

443
444

Table 1. Characteristics of the respondents with LBP by unweighted and weighted numbers, Musculoskeletal Disease Survey 2015

Sociodemographic characteristics	Unweighted sample (n=1,843) number (percent)	Weighted* sample (N=94,794,164) number (percent)
Total	1843 (100.0)	94,794,164 (100.0)
Sex		
Men	892 (48.4)	41,553,976 (43.8)
Women	951 (51.6)	53,240,188 (56.2)
Residence		
Urban	716 (38.9)	27,772,657 (29.3)
Rural	1127 (61.2)	67,021,507 (70.7)
Age, years		
18-34	711 (38.6)	41,343,470 (43.6)
35-54	774 (42.0)	35,278,850 (37.2)
55-99	358 (19.4)	18,171,844 (19.2)

* Weighted to projected population of Bangladesh from 2001 Population Census Frame of Bangladesh Bureau of Statistics.

445

446

For peer review only - http://bmjopen.bmj.com/site/about/guidelines.xhtml

Table 2. Weighted prevalence of low back pain by sociodemographic characteristics in Bangladesh, Musculoskeletal Disease Survey 2015

Sociodemographic characteristics	Total	Men	Women
	Percent (95% CI)	Percent (95% CI)	
Overall	18.5 (11.8–25.2)	13.1 (6.4–19.9)	22.7 (15.3–30.2)
Overall (age-standardized)*	19.4 (14.0–24.8)	14.0 (8.7–19.3)	27.2 (19.3–35.1)
Age in years			
18-34	10.5 (5.7–15.4)	5.2 (1.3–9.0)	13.5 (7.2–19.9)
35-54	23.1 (15.3–30.9)	18.8 (8.7–28.8)	26.7 (18.3–35.1)
55-99	27.8 (16.1–39.5)	15.7 (6.7–24.7)	44.5 (23.9–65.1)
Occupation			
Homemaker	23.6 (15.9–31.2)	–	23.6 (15.9–31.2)
Laborer†	18.2 (9.4–27.0)	17.9 (8.6–27.3)	21.2 (6.2–36.3)
Business professional	9.6 (1.9–17.2)	9.8 (1.9–17.6)	–
Service holder	10.7 (1.2–20.1)	10.3 (0.4–20.2)	–
Others†	13.9 (4.1–23.6)	8.6 (1.7–15.5)	20.3 (6.0–34.6)
Education			
No formal education (0)	31.3 (22.3–40.4)	20.3 (13.4–27.2)	37.4 (24.3–50.5)
Any primary education (1-5)	13.3 (6.7–19.9)	12.1 (3.9–20.3)	14.4 (7.8–21.0)
Any secondary education (6-10)	14.9 (7.4–22.3)	12.0 (2.4–21.6)	17.5 (9.9–25.0)
Above secondary (≥11 years)	6.9 (3.8–10.1)	–	8.0 (3.7–12.3)
Married‡	19.6 (12.4–26.7)	14.1 (6.7–21.4)	23.7 (15.7–31.7)
Wealth index quartile§			
1st	23.5 (13.9–33.0)	19.5 (11.2–27.8)	25.5 (12.9–38.2)
2nd	19.8 (8.4–31.2)	17.3 (3.7–30.9)	21.6 (10.3–32.9)
3rd	14.1 (7.6–20.5)	8.3 (2.3–14.2)	19.8 (11.7–28.0)
4th	16.6 (9.3–23.8)	10.3 (3.2–17.4)	23.1 (11.5–34.7)
Residence			
Urban	14.6 (9.1–20.0)	9.8 (3.9–15.6)	17.7 (8.5–26.8)
Rural	20.2 (10.5–29.8)	14.3 (4.8–23.9)	25.1 (14.7–35.4)
Strenuous physical activity‖	17.1 (4.3–29.8)	17.2 (4.0–30.4)	–
History of physical trauma	24.5 (14.2–34.7)	24.6 (13.0–36.2)	24.4 (7.9–40.9)

All values are percent (95% confidence interval). Weighted percentages shown are calculated from Census 2001 Population Frame by Bangladesh Bureau of Statistics to reflect projected population of Bangladesh (N=94,794,164).

Bold face values denote statistically significant higher prevalence in women compared to men.

* Standardised for WHO World Population 2000-2020.

† Labourer includes: farmer, daily worker, rickshaw puller, garments worker, field worker and others. Other occupations: retired, weaver and housekeeper

‡ Includes currently married, divorced, separated and widowed.

§ The wealth index was constructed using principal component analysis out of a list of 20 household assets (see Methods section for details);

‖ Fourth quartile of the MET-minutes distribution of work-related physical activity. Commutation and leisure time physical activities were not considered because these were negligible contributors.

– 95% confidence interval not reported as number of respondents are <25.

For peer review only - http://bmjopen.bmj.com/site/about/guidelines.xhtml

450

Table 3. Socioeconomic characteristics of patients of low back pain in Bangladesh, Musculoskeletal Disease Survey 2015

Sociodemographic characteristics	Total	Men	Women
	Weighted percentage (95% confidence interval)		
Age in years			
Mean (95% confidence interval)	45.3 (43.0–47.7)	48.3 (45.8–50.9)	44.0 (41.0–47.0)
18–34	24.8 (18.8–30.7)	14.1 (6.9–21.3)	29.6 (21.3–37.9)
35–54	46.4 (40.6–52.2)	55.6 (40.3–70.9)	42.3 (34.4–50.2)
55–99	28.8 (22.5–35.1)	30.3 (17.0–43.7)	28.1 (22.2–34.0)
Occupation			
Homemaker	57.4 (48.2–66.6)	–	83.2 (72.0–94.5)
Laborer†	20.4 (13.6–27.2)	59.7 (38.8–80.5)	2.7 (0.1–5.4)
Business professional	5.2 (1.2–9.1)	16.6 (5.0–28.2)	–
Service holder	–	–	–
Others†	13.5 (2.7–24.3)	14.8 (2.8–26.8)	13.0 (1.5–24.4)
Education			
No formal education (0)	53.2 (41.6–64.9)	39.1 (24.3–54.0)	59.6 (46.9–72.3)
Any primary education (1-5)	17.2 (13.8–20.5)	23.9 (17.1–42.6)	14.1 (10.1–18.1)
Any secondary education (6-10)	24.1 (15.9–32.4)	29.9 (17.1–42.6)	21.6 (13.1–30.1)
Above secondary (≥11 years)	5.5 (1.0–9.9)	–	–
Married‡	97.8 (94.8–100.8)	96.8 (92.2–101.3)	98.3 (95.5–101.1)
Wealth index quartile§			
1st	33.2 (22.6–43.9)	30.1 (18.8–41.4)	34.7 (21.7–47.7)
2nd	25.5 (14.8–36.2)	29.5 (11.5–47.5)	23.7 (14.6–32.7)
3rd	19.7 (11.0–28.4)	18.5 (6.7–30.3)	20.3 (10.5–30.1)
4th	21.6 (9.2–33.9)	21.9 (9.4–34.5)	21.4 (7.1–35.7)
Rural residence	77.0 (55.9–98.0)	80.3 (60.2–100.4)	75.5 (52.8–98.1)

* All values are percent (95% confidence interval) unless stated otherwise. Weighted percentages shown are calculated from Census 2001 Population Frame by Bangladesh Bureau of Statistics to reflect projected population of Bangladesh (N=94,794,164).

† Labourer include: farmer, daily worker, rickshaw puller, garments worker, field worker and others. Other occupations: retired, weaver and housekeeper etc.

‡ Includes currently married, divorced, separated and widowed.

§ The wealth index was constructed using principal component analysis out of a list of 20 household assets (see Methods section for details);

- numbers are low

451

For peer review only - http://bmjopen.bmj.com/site/about/guidelines.xhtml

Table 4: Odds ratios of risk factors for low back pain comapred with no musculoskeletal disorders in Bangladeshi adults, Musculoskeletal Disease Survey 2015

Factors	Odds ratio (95% confidence interval)	
	**Unadjusted**	**Adjusted**
Age group, years	3.2 (2.5–4.2)**	2.4 (1.7–3.4)**
(35–99=1, 18–34=0)	1.0	1.0
Sex	2.1 (1.3–3.3)**	2.2 (1.5–3.3)**
(woman=1, man=0)	1.0	1.0
Labourer†	0.9 (0.7–1.3)	-
(yes=1, no=0)	1.0	-
No formal education	3.5 (2.5–5.0)**	2.3 (1.6–3.3)**
(yes=1, no=0)	1.0	1.0
Low wealth index	1.6 (1.1–2.3)**	1.0 (0.6–1.5)
(yes=1, no=0)	1.0	1.0
Strenuous physical activity‡	0.8 (0.4–1.6)	-
(yes=1, no=0)	1.0	-
Obesity (body mass index ≥25 Kg/m^2)	1.3 (0.8–2.1)	-
(yes=1, no=0)	1.0	-
History of physical trauma	1.8 (1.1–3.2)*	1.6 (0.9–2.8)
(yes=1, no=0)	1.0	1.0
Current tobacco user	1.1 (0.8–1.6)	-
(yes=1, no=0)	1.0	-
Hypertension	2.3 (1.3–4.0)**	1.7 (1.1–2.6)*
(yes=1, no=0)	1.0	1.0
Diabetes mellitus	1.0 (0.5–1.7)	-
(yes=1, no=0)	1.0	-

LBP: low back pain; MSD: musculoskeletal disorder
* $P<0.05$, ** $P<0.01$

† Labourer includes: farmer, daily worker, rickshaw puller, garments worker, field worker and others.

‡ Fourth quartile of the MET-minutes distribution of work-related physical activity. Commutation and leisure time physical activities were not considered because these were negligible contributors.

For peer review only - http://bmjopen.bmj.com/site/about/guidelines.xhtml

Figures

Fig. 1 Flow chart on the selection of low back pain patients from the national survey on musculoskeletal conditions in Bangladesh (2015) adapted from Ahmad Zahid-Al-Quadir *et al* [21]

* Eight divisions from Sept 2015. Randomly 15 districts were selected out of 64.
** PPS indicates population proportion to size.
*** Out-migration, broken house, locked house, no tenant, out of home, refusal. Two recall visits were done if the selected house was locked and the person chosen was not available at home at the time of the interviewer's visit. In case of non-participation after the second recall visit, the targeted household/individual was declared non-respondents.

DRAFT
For peer review only

470 **Fig. 2 Prevalence of LBP in different (Unadjusted overall prevalence (percent, 95%**
471 **confidence interval) of low back pain and in) associated co-conditions (n=343)**
472

*Diabetes was defined as random capillary glucose level ≥11.1 or on medication for diabetes

†Obesity is defined as, body mass index ≥25 kg/m^2;

‡Hypertension was defined as systolic blood pressure ≥140 or diastolic blood pressure ≥90 or medication for hypertension;

473
474

DRAFT

For peer review only

475 **References**

[1] Hoy D, Bain C, Williams G, et al. A systematic review of the global prevalence of low back pain. Arthritis Rheum. 2012 Jun;64(6):2028-37. doi: 10.1002/art.34347.

[2] Dionne CE, Dunn KM, Croft PR, et al. A consensus approach toward the standardization of back pain definitions for use in prevalence studies. Spine (Phila Pa 1976). 2008 Jan 1;33(1):95-103. doi: 10.1097/BRS.0b013e31815e7f94.

[3] Deyo RA, Tsui-Wu YJ. Descriptive epidemiology of low-back pain and its related medical care in the United States. Spine (Phila Pa 1976). 1987 Apr;12(3):264-8. doi: 10.1097/00007632-198704000-00013.

[4] Balagué F, Mannion AF, Pellisé F, et al. Non-specific low back pain. Lancet. 2012 Feb 4;379(9814):482-91. doi: 10.1016/S0140-6736(11)60610-7.

[5] Andersson GB. Epidemiological features of chronic low-back pain. Lancet. 1999 Aug 14;354(9178):581-5. doi: 10.1016/S0140-6736(99)01312-4.

[6] Fatoye F, Gebrye T, Odeyemi I. Real-world incidence and prevalence of low back pain using routinely collected data. Rheumatol Int. 2019 Apr;39(4):619-626. doi: 10.1007/s00296-019-04273-0.

[7] Hartvigsen J, Hancock MJ, Kongsted A, et al. Lancet Low Back Pain Series Working Group. What low back pain is and why we need to pay attention. Lancet. 2018 Jun 9;391(10137):2356-2367. doi: 10.1016/S0140-6736(18)30480-X.

[8] Parreira P, Maher CG, Steffens D, et al. Risk factors for low back pain and sciatica: an umbrella review. Spine J. 2018 Sep;18(9):1715-1721. doi: 10.1016/j.spinee.2018.05.018.

[9] Lötters F, Burdorf A. Prognostic factors for duration of sickness absence due to musculoskeletal disorders. The Clinical Journal of Pain. 2006 Feb;22(2):212-221. DOI: 10.1097/01.ajp.0000154047.30155.72.

For peer review only - http://bmjopen.bmj.com/site/about/guidelines.xhtml

[10] Guo HR, Tanaka S, Halperin WE, et al. Back pain prevalence in US industry and estimates of lost workdays. Am J Public Health. 1999 Jul;89(7):1029-35. doi: 10.2105/ajph.89.7.1029.

[11] Cassidy JD, Carroll LJ, Côté P. The Saskatchewan health and back pain survey. The prevalence of low back pain and related disability in Saskatchewan adults. Spine. 1998 Sep 1;23(17):1860-6; discussion 1867. doi: 10.1097/00007632-199809010-00012.

[12] Harreby M, Kjer J, Hesselsøe G, Neergaard K. Epidemiological aspects and risk factors for low back pain in 38-year-old men and women: a 25-year prospective cohort study of 640 school children. Eur Spine J. 1996;5(5):312-8. doi: 10.1007/BF00304346.

[13] Waxman R, Tennant A, Helliwell P. A prospective follow-up study of low back pain in the community. Spine. 2000 Aug 15;25(16):2085-90. doi: 10.1097/00007632-200008150-00013.

[14] Biglarian A, Seifi B, Bakhshi E, et al. Low back pain prevalence and associated factors in Iranian population: findings from the national health survey. Pain Res Treat. 2012;2012:653060. doi: 10.1155/2012/653060.

[15] Gupta G, Nandini N. Prevalence of low back pain in non working rural housewives of Kanpur, India. Int J Occup Med Environ Health. 2015;28(2):313-20. doi: 10.13075/ijomeh.1896.00299.

[16] Ahdhi GS, Subramanian R, Saya GK, et al. Prevalence of low back pain and its relation to quality of life and disability among women in rural areas of Puducherry, India. Indian J Pain [serial online] 2016 [cited 2021 Apr 18];30:111-5. Available from: https://www.indianjpain.org/text.asp?2016/30/2/111/186467.

[17] Bansal D, Asrar MM, Ghai B, et al. Prevalence and Impact of Low Back Pain in a Community-Based Population in Northern India. Pain Physician. 2020;23(4):E389-E98.

[18] Haq SA, Darmawan J, Islam MN, et al. Prevalence of rheumatic diseases and associated outcomes in rural and urban communities in Bangladesh: a COPCORD study. J Rheumatol. 2005 Feb;32(2):348-53. PMID: 15693098.

For peer review only - http://bmjopen.bmj.com/site/about/guidelines.xhtml

[19] Zahid-Al-Quadir, A., Zaman, M.M., Ahmed, S. et al. Prevalence of musculoskeletal conditions and related disabilities in Bangladeshi adults: a cross-sectional national survey. BMC Rheumatol 4, 69 (2020). https://doi.org/10.1186/s41927-020-00169-w

[20] Bangladesh Bureau of Statistics. Population and housing census 2001, preliminary result. Dhaka: Bangladesh Bureau of Statistics; 2001. Accessed 20 Apr 2020. http://bbs.portal.gov.bd.

[21] Islam, ME, Ahmed, S, Shahin, MA, et al. Cross-cultural adaptation and validation of revised WHO-ILAR-COPCORD core English questionnaire translated to Bangla. Int J Rheum Dis. 2021; 00: 1– 14. https://doi.org/10.1111/1756-185X.14176

[22] Beaton DE, Bombardier C, Guillemin F, Ferraz MB. Guidelines for the process of cross-cultural adaptation of self-report measures. Spine (Phila Pa 1976). 2000 Dec 15;25(24):3186-91. doi: 10.1097/00007632-200012150-00014.

[23] Chassany O, Marquis P, Scherrer B, Read NW, Finger T, Bergmann JF, Fraitag B, Geneve J, Caulin C. Validation of a specific quality of life questionnaire for functional digestive disorders. Gut. 1999 Apr;44(4):527-33. doi: 10.1136/gut.44.4.527.

[24] van Tulder M, Becker A, Bekkering T, et al. COST B13 Working Group on Guidelines for the Management of Acute Low Back Pain in Primary Care. Chapter 3. European guidelines for the management of acute nonspecific low back pain in primary care. Eur Spine J. 2006 Mar;15 Suppl 2(Suppl 2):S169-91. doi: 10.1007/s00586-006-1071-2.

[25] Zaman MM, Rahman MM, Rahman MR, et al. Prevalence of risk factors for non-communicable diseases in Bangladesh: Results from STEPS survey 2010. Indian J Public Health. 2016 Jan-Mar;60(1):17-25. doi: 10.4103/0019-557X.177290.

[26] Islam N, Baron Basak T, OudeVoshaar MA, et al. Cross-cultural adaptation and validation of a Bengali Health Assessment Questionnaire for use in rheumatoid arthritis patients. Int J Rheum Dis. 2013 Aug;16(4):413-7. doi: 10.1111/1756-185X.12032.

DRAFT
For peer review only

[27] Misra A. Ethnic-Specific Criteria for Classification of Body Mass Index: A Perspective for Asian Indians and American Diabetes Association Position Statement. Diabetes Technol Ther. 2015 Sep;17(9):667-71. doi: 10.1089/dia.2015.0007.

[28] Hakim, F., Bhuiyan, R., Akter, M. K., Mohit, M. A., Alam, M. F., Karim, M. R., & Zaman, M. M. (2022). Weighting National Survey Data in Bangladesh: Why, How and Which weight? Weighting survey data. Bangladesh Medical Research Council Bulletin, 47(2), 118–126. https://doi.org/10.3329/bmrcb.v47i2.57769

[29] Ahmad OB, Boschi-Pinto C, Lopez AD, et al. Age Standardization of Rates: A New WHO Standard. GPE Discussion Paper Series: No31 Geneva, Switzerland: World Health Organization, 2001.

[30] WMA Declaration of Helsinki - ethical principles for medical research involving human respondents. [Cited on: 2020 Aug 29] Available from: https://www.wma.net/policies-post/wma-declaration-of-helsinki-ethical-principlesfor-medical-research-involving-human-respondents/.

[31] Dutmer AL, Schiphorst Preuper HR, Soer R, et al. Personal and Societal Impact of Low Back Pain: The Groningen Spine Cohort. Spine (Phila Pa 1976). 2019 Dec 15;44(24):E1443-E1451. doi: 10.1097/BRS.0000000000003174.

[32] Al-Awadhi AM, Olusi SO, Al-Saeid K, et al. Incidence of musculoskeletal pain in adult Kuwaitis using the validated Arabic version of the WHO-ILAR COPCORD Core Questionnaire. Annals of Saudi Medicine. 2005 Nov-Dec;25(6):459-462. DOI: 10.5144/0256-4947.2005.459.

[33] Aldeen Sarakbi H, Alsaed O, Hammoudeh M, et al.. Epidemiology of musculoskeletal complaints and diseases in Qatar: A cross-sectional study. Qatar Med J. 2020 Nov 12;2020(2):29. doi: 10.5339/qmj.2020.29.

For peer review only - http://bmjopen.bmj.com/site/about/guidelines.xhtml

[34] Bansal D, Asrar MM, Ghai B, et al. Prevalence and Impact of Low Back Pain in a Community-Based Population in Northern India. Pain Physician. 2020 Jul;23(4):E389-E398. PMID: 32709185.

[35] Veerapen K, Wigley RD, Valkenburg H. Musculoskeletal pain in Malaysia: a COPCORD survey. J Rheumatol. 2007 Jan;34(1):207-13. PMID: 17216688.

[36] Sany SA, Tanjim T and Hossain MI. Low back pain and associated risk factors among medical students in Bangladesh: a cross-sectional study. F1000Research. 2021 July; 10:698. Doi: 10.12688/f1000research.55151.1.

[37] Ali M, Ahsan GU, Hossain A. Prevalence and associated occupational factors of low back pain among the bank employees in Dhaka City. J Occup Health. 2020 Jan; 62(1):e12131. doi: 10.1002/1348-9585.12131.

[38] Mondal R, Sarker RC, Akter S, Banik PC, Baroi SK. Prevalence of low back pain and its associated factors among physiotherapists in Dhaka city of Bangladesh in 2016. Journal of Occupational Health and Epidemiology. 2018. 7:70–4. Doi: 10.29252/johe.7.2.70.

[39] GBD 2017 Disease and Injury Incidence and Prevalence Collaborators. Global, regional, and national incidence, prevalence, and years lived with disability for 354 diseases and injuries for 195 countries and territories, 1990-2017: a systematic analysis for the Global Burden of Disease Study 2017. Lancet. 2018 Nov 10;392(10159):1789-1858. doi: 10.1016/S0140-6736(18)32279-7.

[40] Hoy D, Brooks P, Blyth F, et al. The Epidemiology of low back pain. Best Practice & Research Clinical Rheumatology. 2010 Dec;24(6):769–81. doi: 10.1016/j.berh.2010.10.002.

[41] Loney PL, Stratford PW. The prevalence of low back pain in adults: a methodological review of the literature. Phys Ther. 1999 Apr;79(4):384-96. PMID: 10201544.

[42] Manogharan S, Kongsted A, Ferreira ML, et al. Do older adults with chronic low back pain differ from younger adults in regards to baseline characteristics and prognosis? Eur J Pain (United Kingdom). 2017;21(5):866–73.

For peer review only - http://bmjopen.bmj.com/site/about/guidelines.xhtml

[43] Ganesan S, Acharya AS, Chauhan R, Acharya S. Prevalence and Risk Factors for Low Back Pain in 1,355 Young Adults: A Cross-Sectional Study. Asian Spine J. 2017 Aug;11(4):610-617. doi: 10.4184/asj.2017.11.4.610.

[44] Yue P, Liu F, Li L. Neck/shoulder pain and low back pain among school teachers in China, prevalence and risk factors. BMC Public Health. 2012 Sep 14;12(1). doi: 10.1186/1471-2458-12-789.

[45] Craft RM, Mogil JS, Aloisi AM. Sex differences in pain and analgesia: the role of gonadal hormones. European Journal of Pain. 2004 Oct;8(5):397–411. doi: 10.1016/j.ejpain.2004.01.003

[46] Berkley KJ. Sex differences in pain. Behav Brain Sci. 1997 Sep;20(3):371–80. doi: 10.1017/s0140525x97221485.

[47] Keogh E. Sex Differences in Pain. Reviews in Pain. 2008 Dec;2(2):4–7. doi: 10.1177/204946370800200203.

[48] Leboeuf-Yde C. Body Weight and Low Back Pain. Spine. 2000 Jan;25(2):226. doi: 10.1097/00007632-200001150-00015.

[49] Webb R, Brammah T, Lunt M, et al. Prevalence and Predictors of Intense, Chronic, and Disabling Neck and Back Pain in the UK General Population. Spine. 2003 Jun;28(11):1195–202. doi: 10.1097/01.BRS.0000067430.49169.01.

[50] Koyanagi A, Stickley A, Garin N, Miret M, Ayuso-Mateos JL, Leonardi M, Koskinen S, Galas A, Haro JM. The association between obesity and back pain in nine countries: a cross-sectional study. BMC Public Health. 2015 Feb 11;15:123. doi: 10.1186/s12889-015-1362-9.

[51] Shmagel A, Foley R, Ibrahim H. Epidemiology of Chronic Low Back Pain in US Adults: Data From the 2009-2010 National Health and Nutrition Examination Survey. Arthritis Care & Research. 2016 Oct 26;68(11):1688–94. doi: 10.1002/acr.22890.

For peer review only - http://bmjopen.bmj.com/site/about/guidelines.xhtml

[52] Deyo RA, Tsui-wu YJ. Descriptive Epidemiology of Low-back Pain and Its Related Medical Care in the United States. Spine. 1987 Apr;12(3):264–8. doi: 10.1097/00007632-198704000-00013.

[53] Rubin DI. Epidemiology and Risk Factors for Spine Pain. Neurologic Clinics. 2007 May;25(2):353–71. doi: 10.1016/j.ncl.2007.01.004.

[54] Frymoyer JW, Pope MH, Costanza MC, et al. Epidemiologic Studies of Low-Back Pain. Spine. 1980 Sep;5(5):419–23. doi: 10.1097/00007632-198009000-00005.

[55] Coggon D, Ntani G, Palmer KT, et al. Drivers of international variation in prevalence of disabling low back pain: Findings from the Cultural and Psychosocial Influences on Disability study. European journal of pain. 2018 Jun 27;23(1):35–45. doi: 10.1002/ejp.1255.

[56] Hestbaek L, Korsholm L, Leboeuf-Yde C, Kyvik KO. Does socioeconomic status in adolescence predict low back pain in adulthood? A repeated cross-sectional study of 4,771 Danish adolescents. Eur Spine J. 2008 Dec;17(12):1727-34. doi: 10.1007/s00586-008-0796-5.

[57] Harris IA, Young JM, Rae H, et al. Factors associated with back pain after physical injury: a survey of consecutive major trauma patients. Spine (Phila Pa 1976). 2007 Jun 15;32(14):1561-5. doi: 10.1097/BRS.0b013e318067dce8.

[58] Samartzis D, Bow C, Karppinen J, Luk KDK, Cheung BMY, Cheung KMC. Hypertension is Independently Associated with Lumbar Disc Degeneration: A Large-Scale Population-Based Study. Global Spine Journal, vol. 4, no. 1_suppl, May 2014, p. s-0034-1376579-s-0034-1376579. doi:10.1055/s-0034-1376579.

[59] Leino-Arjas P, Solovieva S, Kirjonen J, Reunanen A, Riihimäki H. Cardiovascular risk factors and low-back pain in a long-term follow-up of industrial employees. Scand J Work Environ Health. 2006 Feb;32(1):12-9. doi: 10.5271/sjweh.971.

For peer review only - http://bmjopen.bmj.com/site/about/guidelines.xhtml

[60] Bae Y-H, Shin J-S, Lee J, et al. Association between Hypertension and the Prevalence of Low Back Pain and Osteoarthritis in Koreans: A Cross-Sectional Study. Fuchs FD, editor. PLoS ONE. 2015 Sep 22;10(9):e0138790. doi: 10.1371/journal.pone.0138790.

[61] Rahman MA, Khan MA, Akter MR, Islam MA, Khan MK. Proportion of Low Back Pain among Physicians Working in a Tertiary Level Hospital of Bangladesh. Mymensingh Medical Journal: MMJ. 2021 Apr 1;30(2):262-6. PMID: 33830101

DRAFT

For peer review only

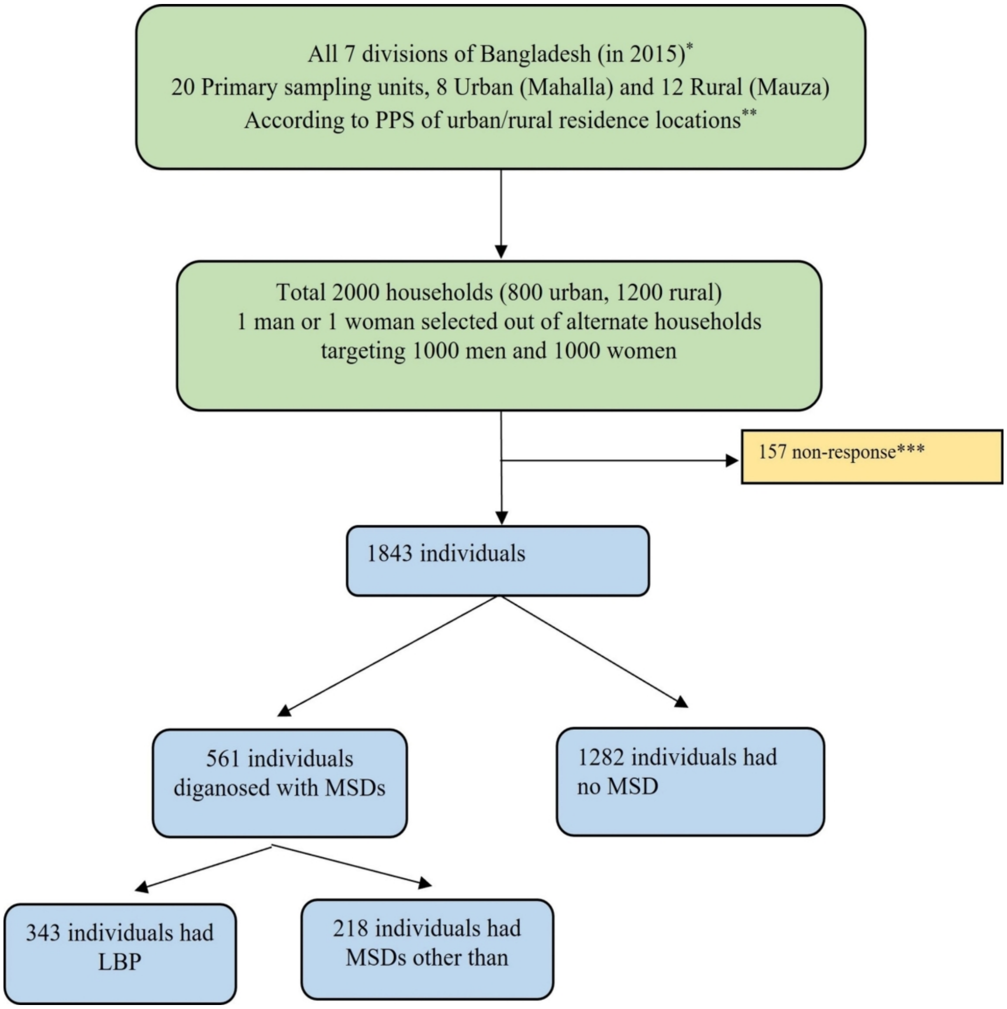

Caption:
Fig. 1 Flow chart on the selection of low back pain patients from the national survey on musculoskeletal conditions in Bangladesh (2015) adapted from Ahmad Zahid-Al-Quadir et al 21
Legend:
* Eight divisions from Sept 2015. Randomly 15 districts were selected out of 64.
** PPS indicates population proportion to size.
*** Out-migration, broken house, locked house, no tenant, out of home, refusal. Two recall visits were done if the selected house was locked and the person chosen was not available at home at the time of the interviewer's visit. In case of non-participation after the second recall visit, the targeted household/individual was declared non-respondents.

190x190mm (300 x 300 DPI)

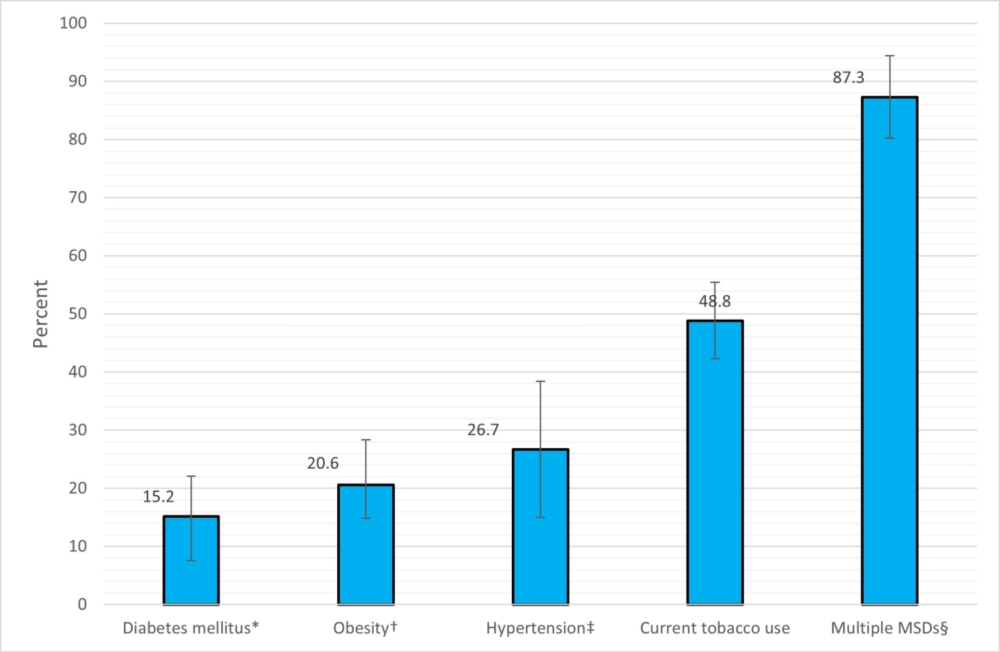

Caption:
Fig. 2 Prevalence of LBP in different (Unadjusted overall prevalence (percent, 95% confidence interval) of low back pain and in) associated co-conditions (n=343)
Legend:
*Diabetes was defined as random capillary glucose level ≥11.1 or on medication for diabetes
†Obesity is defined as, body mass index ≥25 kg/m2;
‡Hypertension was defined as systolic blood pressure ≥140 or diastolic blood pressure ≥90 or medication for hypertension

597x389mm (300 x 300 DPI)

STROBE 2007 (v4) Statement—Checklist of items that should be included in reports of *cross-sectional studies*

Section/Topic	Item #	Recommendation	Reported on page #
**Title and abstract**	1	(*a*) Indicate the study's design with a commonly used term in the title or the abstract	1
		(*b*) Provide in the abstract an informative and balanced summary of what was done and what was found	2
**Introduction**			
Background/rationale	2	Explain the scientific background and rationale for the investigation being reported	4, 5
Objectives	3	State specific objectives, including any prespecified hypotheses	5
**Methods**			
Study design	4	Present key elements of study design early in the paper	5
Setting	5	Describe the setting, locations, and relevant dates, including periods of recruitment, exposure, follow-up, and data collection	5, 6
Participants	6	(*a*) Give the eligibility criteria, and the sources and methods of selection of participants	5
Variables	7	Clearly define all outcomes, exposures, predictors, potential confounders, and effect modifiers. Give diagnostic criteria, if applicable	6
Data sources/ measurement	8*	For each variable of interest, give sources of data and details of methods of assessment (measurement). Describe comparability of assessment methods if there is more than one group	5, 6
Bias	9	Describe any efforts to address potential sources of bias	5, 6
Study size	10	Explain how the study size was arrived at	5
Quantitative variables	11	Explain how quantitative variables were handled in the analyses. If applicable, describe which groupings were chosen and why	6
Statistical methods	12	(*a*) Describe all statistical methods, including those used to control for confounding	7
		(*b*) Describe any methods used to examine subgroups and interactions	7
		(*c*) Explain how missing data were addressed	7
		(*d*) If applicable, describe analytical methods taking account of sampling strategy	7
		(*e*) Describe any sensitivity analyses	Not applicable
**Results**			

For peer review only

Participants	13*	(a) Report numbers of individuals at each stage of study—eg numbers potentially eligible, examined for eligibility, confirmed eligible, included in the study, completing follow-up, and analysed	5, 22
		(b) Give reasons for non-participation at each stage	19
		(c) Consider use of a flow diagram	19
Descriptive data	14*	(a) Give characteristics of study participants (eg demographic, clinical, social) and information on exposures and potential confounders	7, 8
		(b) Indicate number of participants with missing data for each variable of interest	Not applicable
Outcome data	15*	Report numbers of outcome events or summary measures	8, 9
Main results	16	(*a*) Give unadjusted estimates and, if applicable, confounder-adjusted estimates and their precision (eg, 95% confidence interval). Make clear which confounders were adjusted for and why they were included	9
		(*b*) Report category boundaries when continuous variables were categorized	6
		(*c*) If relevant, consider translating estimates of relative risk into absolute risk for a meaningful time period	Not applicable
Other analyses	17	Report other analyses done—eg analyses of subgroups and interactions, and sensitivity analyses	Not applicable
**Discussion**			
Key results	18	Summarise key results with reference to study objectives	10
Limitations	19	Discuss limitations of the study, taking into account sources of potential bias or imprecision. Discuss both direction and magnitude of any potential bias	3
Interpretation	20	Give a cautious overall interpretation of results considering objectives, limitations, multiplicity of analyses, results from similar studies, and other relevant evidence	11, 12
Generalisability	21	Discuss the generalisability (external validity) of the study results	13
**Other information**			
Funding	22	Give the source of funding and the role of the funders for the present study and, if applicable, for the original study on which the present article is based	14

*Give information separately for cases and controls in case-control studies and, if applicable, for exposed and unexposed groups in cohort and cross-sectional studies.

Note: An Explanation and Elaboration article discusses each checklist item and gives methodological background and published examples of transparent reporting. The STROBE checklist is best used in conjunction with this article (freely available on the Web sites of PLoS Medicine at http://www.plosmedicine.org/, Annals of Internal Medicine at http://www.annals.org/, and Epidemiology at http://www.epidem.com/). Information on the STROBE Initiative is available at www.strobe-statement.org.

For peer review only

BMJ Open

Low Back Pain in the Bangladeshi Adult Population: A Cross-sectional National Survey

Journal:	*BMJ Open*
Manuscript ID	bmjopen-2021-059192.R2
Article Type:	Original research
Date Submitted by the Author:	17-Aug-2022

Complete List of Authors:	Momen Majumder, Muhammad Shoaib; Bangabandhu Sheikh Mujib Medical University, Rheumatology<br>Hakim, Ferdous; World Health Organization Bangladesh<br>Bandhan, Iftekhar ; Bangabandhu Sheikh Mujib Medical University, Rheumatology<br>Razzaque, Mohammad Abdur; Chittagong Medical College, Medicine<br>Zahid-Al-Quadir, Ahmad ; Sylhet MAG Osmani Medical College, Medicine<br>Ahmed, Shamim; Bangabandhu Sheikh Mujib Medical University<br>Choudhury, Minhaj; Bangabandhu Sheikh Mujib Medical University, Rheumatology<br>Haq, Syed; Bangabandhu Sheikh Mujib Medical University, Rheumatology<br>Zaman, MM; WHO Bangladesh

**Primary Subject Heading**:	Epidemiology
Secondary Subject Heading:	Rheumatology
Keywords:	EPIDEMIOLOGY, RHEUMATOLOGY, PUBLIC HEALTH, PREVENTIVE MEDICINE

SCHOLARONE™ Manuscripts

I, the Submitting Author has the right to grant and does grant on behalf of all authors of the Work (as defined in the below author licence), an exclusive licence and/or a non-exclusive licence for contributions from authors who are: i) UK Crown employees; ii) where BMJ has agreed a CC-BY licence shall apply, and/or iii) in accordance with the terms applicable for US Federal Government officers or employees acting as part of their official duties; on a worldwide, perpetual, irrevocable, royalty-free basis to BMJ Publishing Group Ltd ("BMJ") its licensees and where the relevant Journal is co-owned by BMJ to the co-owners of the Journal, to publish the Work in this journal and any other BMJ products and to exploit all rights, as set out in our licence.

The Submitting Author accepts and understands that any supply made under these terms is made by BMJ to the Submitting Author unless you are acting as an employee on behalf of your employer or a postgraduate student of an affiliated institution which is paying any applicable article publishing charge ("APC") for Open Access articles. Where the Submitting Author wishes to make the Work available on an Open Access basis (and intends to pay the relevant APC), the terms of reuse of such Open Access shall be governed by a Creative Commons licence – details of these licences and which Creative Commons licence will apply to this Work are set out in our licence referred to above.

Other than as permitted in any relevant BMJ Author's Self Archiving Policies, I confirm this Work has not been accepted for publication elsewhere, is not being considered for publication elsewhere and does not duplicate material already published. I confirm all authors consent to publication of this Work and authorise the granting of this licence.

For peer review only

Low Back Pain in the Bangladeshi Adult Population: A Cross-sectional National Survey

Short Running Head: *Low Back Pain in Bangladesh*

Authors: Muhammad Shoaib Momen Majumder, Ferdous Hakim, Iftekhar Hussain Bandhan, Mohammad Abdur Razzaque, Ahmad Zahid-Al-Quadir, Shamim Ahmed, Minhaj Rahim Choudhury, Syed Atiqul Haq, Mohammad Mostafa Zaman

Author names, highest academic degrees, appointments, Email and ORCID:

Muhammad Shoaib Momen Majumder, FCPS (Medicine), MD (Rheumatology); Department of Rheumatology, Bangabandhu Sheikh Mujib Medical University (Ringgold ID 74464), Bangladesh, Email: momen.shoaib@gmail.com, ORCID ID: https://orcid.org/0000-0002-7901-0847;

Ferdous Hakim, MPH (Epidemiology), Research and Publication, World Health Organization Bangladesh (Ringgold ID 215134), Email: hakimf@who.int, ORCID: https://orcid.org/0000-0003-2376-3978;

Iftekhar Hussain Bandhan, MD (Rheumatology), Department of Rheumatology, Bangabandhu Sheikh Mujib Medical University (Ringgold ID 74464), Bangladesh, Email: ibandhan@gmail.com, ORCID ID: https://orcid.org/0000-0002-3800-6155;

Mohammad Abdur Razzaque, MD (Rheumatology), Register, Department of Medicine, Chittagong Medical College (Ringgold ID 467859), Bangladesh, Email: rhrazzaq@gmail.com, ORCID ID: https://orcid.org/0000-0002-2395-542X;

Ahmad Zahid-Al-Quadir, MD (Rheumatology), Department of Medicine, Sylhet MAG Osmani Medical College (Ringgold ID 469196), Email: drzahidssmc@gmail.com, https://orcid.org/0000-0003-4178-6487;

Shamim Ahmed, FCPS (Medicine), Associate Professor of Rheumatology, Bangabandhu Sheikh Mujib Medical University (Ringgold ID 74464), Dhaka, Bangladesh, Email: dr_shamim_ahmed@yahoo.com;

Minhaj Rahim Choudhury, MD, FCPS (Medicine) Professor of Rheumatology, Bangabandhu Sheikh Mujib Medical University (Ringgold ID 74464), Dhaka, Bangladesh, Email: drminhaj11@gmail.com, ORCID ID: https://orcid.org/0000-0002-8695-5240;

Syed Atiqul Haq, FCPS (Medicine), MD, FRCP, Professor of Rheumatology, Bangabandhu Sheikh Mujib Medical University (Ringgold ID 74464), Dhaka, Bangladesh, Email: haqsyedatiqul@gmail.com, ORCID ID: https://orcid.org/0000-0003-4154-7283

Mohammad Mostafa Zaman, PhD (Epidemiology), Advisor, Research and Publication, World Health Organization Bangladesh (Ringgold ID 215134), Email: zamanm@who.int, ORCID ID: https://orcid.org/0000-0002-1736-1342;

Counts: Number of words (abstract): 263, number of words (body): 3471, number of references: 61, number of tables: 04, number of figures: 02

Name, address, email of the author responsible for correspondence:

For peer review only

For peer review only - http://bmjopen.bmj.com/site/about/guidelines.xhtml

Muhammad Shoaib Momen Majumder, FCPS (Medicine), MD (Rheumatology); Department of Rheumatology, Bangabandhu Sheikh Mujib Medical University (Ringgold ID 74464), Bangladesh, Email: momen.shoaib@gmail.com, ORCID ID: https://orcid.org/0000-0002-7901-0847

ABSTRACT

Objective:

Low back pain (LBP) is a common musculoskeletal disorder. This study aims to determine the residence- and sex-specific prevalence and the factors associated with LBP in Bangladesh.

Methods:

The study subjects (ages ≥18 years) were identified from 20 primary sampling units of the national census following a cross-sectional multi-stage stratified sampling design. We considered the mechanical type of LBP for this study. A Bangla version of the modified Community Oriented Program for Control of Rheumatic Disorders questionnaire was used. A team of trained field workers, rheumatology residents and rheumatologists collected the data. Analysis was done using weighted data.

Results:

Two thousand subjects were approached, but 1843 could be screened. Among them, 561 had musculoskeletal disorders, and 343 were diagnosed with LBP. The weighted prevalence of LBP was 18.5% (95% confidence interval, CI: 11.8–25.2) and age-standardized prevalence of LBP was 19.4% (95% confidence interval, CI: 14.0–24.8), which was higher in women (27.2%, 19.3-35.1) than men (14.0%, 8.7-19.3). The prevalence persistently increased from age group 18-34 years (10.5%, 5.7–15.4) to ≥55 years (27.8%, 16.1–39.5). People with no education had the highest prevalence (31.3%, 22.3–40.4). The prevalence did not differ between urban and rural residential locations. Four factors were significantly associated with LBP: age (adjusted odds ratio (2.4, 95% CI: 1.7–3.4), female sex (2.2, 1.5–3.3), absence of formal education (2.3, 1.6–3.3), and hypertension (1.7, 1.1–2.6).

Conclusion:

For peer review only - http://bmjopen.bmj.com/site/about/guidelines.xhtml

LBP is a common problem in Bangladeshi adults. The factors identified are age, female sex, no formal education, and hypertension. These should be addressed adequately to prevent and treat LBP.

Keywords: Low back pain, factors, Bangladesh, prevalence, cross-sectional survey

Strengths and Limitations

- We report the weighted prevalence of low back pain by sociodemographic characteristics, comorbidities, disability and work loss, and identified factors associated with back pain patients, for the first time in Bangladesh.
- All the diagnoses were made by rheumatology residents and expert rheumatologists in the field.
- Some diagnoses of evolving rheumatological conditions might lack validity because of lack of quality laboratory facilities in the field.
- The sample size calculation is based on combined prevalence of musculoskeletal disorders that warrant cautious interpretation of the results because of inadequate sample size, especially when split into reporting domains.
- Recall period for determining work loss was 12 months which might induce bias.

For peer review only

For peer review only - http://bmjopen.bmj.com/site/about/guidelines.xhtml

INTRODUCTION

Low back pain (LBP) is one of the most frequent medical problems globally [1]. It is defined as pain, stiffness, or muscle tension localized below the costal margin and above the inferior gluteal folds [2]. Up to 84% of adults suffer from LBP at some point in life [3]. The prevalence of chronic LBP is about 23% [4]. Around 11–12% of the population become disabled due to LBP [1]. It causes substantial personal, social and financial burdens globally [1]. In the USA, LBP is the second most frequent cause for a physician consultation [5]. LBP is ranked globally as the topmost cause of disability as it affects mostly working-age people [6]. It accounted for 60.1 million disability-adjusted life-years in 2015 [7]. There was a significant increase of LBP by 54% since 1990, and the highest escalation took place in the low and middle-income countries (LMICs) [7]. Disability from LBP is a primary concern for the LMICs, specially in Bangladesh where manual labour–rickshaw pulling, day laborers, house maids, work exposure to lifting of heavy weight during their day-to-day activities etc.– is common [7]. The scope for job switching is restricted in resource constraint countries.

LBP has multi-sectorial health outcomes like a lower quality of life, poorer self-reported health, depression and more workspace absenteeism [8]. As a result, LBP has become an important cause of sick leave and early retirement among the working population [9]. In the USA, approximately 149 million workdays are lost due to LBP, leading to an estimated loss of 100–200 billion US dollars per year [10]. Non-specific LBP is the commonest of all causes of LBP [4]. Non-specific LBP is defined as LBP not particularly attributable to specific aetiology like malignancy, infection, fracture, inflammatory condition, radiculopathy or cauda equina syndrome [4].

Albeit high in most studies, there is a difference in LBP prevalence in various epidemiological studies. The estimated lifetime prevalence was 84.1% in a Canadian study [11], 70% in Denmark [12], 59% in the UK [13]. In Iran, the prevalence of LBP was 29.3% [14]. The estimated prevalence of LBP in India ranged between 42 and 83% [15, 16]. A recent cross-sectional, community-based, epidemiological study conducted in Northern India yielded an estimated lifetime prevalence of 47% in man 57% in women [17]. A Community Oriented Program for Control of Rheumatic Disorders (COPCORD) survey in Bangladesh published in 2005 showed 6.6%, 9.9%, and 9.2% prevalence of LBP in the rural, urban slum,

For peer review only

For peer review only - http://bmjopen.bmj.com/site/about/guidelines.xhtml

and affluent urban areas, respectively [18]. A cross-sectional national study in Bangladesh in 2015 showed LBP was the top-ranking musculoskeletal disorder (MSD) with a prevalence of 18.6% [19]. We have further analyzed the data from the 2015 study and report the population weighted prevalence according to sociodemographic factors, comorbid conditions, disability and work loss due to LBP, and identify the factors associated with LBP.

METHODS

A detailed description of the methodology is beyond the scope of this article and is described elsewhere [19]. Adults aged 18 years or more comprised the study population through a household level multi-stage stratified cross-sectional survey. The sampling frame was based on the 2001 Bangladesh Census [20]. Based on a point prevalence of MSD and with a design effect of 1.5 and 85% response rate for four reporting domains (man-woman, urban-rural), the calculated sample size was 1,978, which was rounded to 2,000. It was stratified into seven divisions of rural (Mauza) and urban (Mahalla) areas. Twenty (8 urban and 12 rural) primary sampling units (PSUs) were selected. The first 100 households were consecutively included from each PSU, where even numbers were assigned as man and odd numbers as woman households. In each household, the single respondent was identified from a list of eligible household members with the help of a Kish table. Data was collected in November and December of 2015. (**Figure 1**)

A detailed manual was prepared before the training for this survey and was used by all field staff. All investigators and the WHO technical team coordinated and conducted the training. The modified COPCORD questionnaire was the survey tool [21]. The English version of the first part of the questionnaire was translated to Bangla, then adapted according to the guideline of Beaton et al [22], validated by Chassany's Method [23], and administered by the interviewers. Data were collected for six days from each PSU. There were two recall visits to ensure participation. The research physician interviewed the suspected respondents for MSDs. A subject was considered a positive respondent if he/she reported pain in muscles, bones, joints, or any part of the body (musculoskeletal system) during the preceding seven days. Subjects who were taking pain medications like non-steroidal anti-inflammatory drugs (NSAID) or steroids were considered as positive respondent even if they did not report pain on those seven days. All positive respondents were interviewed and examined by the research

For peer review only - http://bmjopen.bmj.com/site/about/guidelines.xhtml

physicians. Internationally accepted criteria were used for the diagnosis of the diseases. For the conditions without any internationally accepted criteria, relevant investigations and clinical judgment of the research physician was used. The final diagnoses were checked and verified by a rheumatologist during their visit to respective PSUs.

LBP group of disorders were operationally defined as mechanical type back pain that included non-specific LBP and lumbar spondylosis. Considering the limitation of differentiating investigation in the field, we did not classify LBP beyond this. LBP duration was classified into three groups: acute: up to 6 weeks, subacute: 6-12 weeks, and chronic that persists beyond 12 weeks [24]. Respondents with pain in the muscles, bones, joints, or any part of the body (musculoskeletal symptom) during the preceding seven days or on pain medication with no pain were considered as positive respondents. The research physicians interviewed and thoroughly examined all 'positive' respondents. Data on physical activity were calculated into metabolic equivalent tasks (MET)-minutes per week using the STEPwise Surveillance of noncommunicable disease risk factors (STEPS) protocol and divided into quartiles [25]. The 4th quartile was labelled as strenuous physical activity.

The study participants were divided into three subgroups as per age in years: 18-34, 35-54 and 55-99. We considered ownership of household asset items (electricity, television, refrigerator, etc.) for constructing wealth index. In addition, the type of main material used for the roof of the main house (cement, tin and katcha such as bamboo/thatched/straw/gunny etc.) was also included in the model. A principal component analysis was used to create standardized factor scores for each of the items. The total scores for the respondents were calculated and categorized those into quartiles for description from one (lowest household wealth) to four (highest household wealth) [19].

A validated Bangla version of the Bangla Health Assessment Questionnaire - Disability Index (HAQ-DI) was used for the disability score. For determining work loss, the recall period was 12 months [26]. Random capillary blood glucose was measured by using glucometer (Accu-Check Germany). Using height (meters) and weight (kilograms) measurements, we calculated BMI [weight (kg)/height (meter)^2]. Waist circumference was measured by horizontally placing a measuring tape above the iliac crest. Diabetes was defined as random blood glucose

For peer review only - http://bmjopen.bmj.com/site/about/guidelines.xhtml

≥11.1 mmol/l or the use of anti-diabetic medications. Obesity was defined as a body mass index of ≥25 kg/meter squared [27].

Statistical analysis:

The survey data were entered in and cleaned using Microsoft Excel. We have weighted [28] the data to reflect population frame of Bangladesh for the year 2015. Base weight–for the sampled population–was calculated using probability of selection of respondents among the eligible number of members of household in a cluster defined by divisions (7), age groups (3) and sexes (2). The base weight was adjusted with non-response weights separately for men and women by three age groups. The final weight was generated after calibration to frame population (2015) in domains by division, sex and age groups. Analysis was done using the final weights.

Statistical analysis was done using Epi Info Version 7.1.5.2 and in SPSS Version 20.0. Continuous variables were categorized before analysis as appropriate. We estimated the prevalence of LBP with 95% confidence intervals. The prevalence was segregated by residence (urban/ rural) and sex (man/ woman). Nonparametric test (Kruskal-Wallis H test) was used to analyze data that were not normally distributed. Whenever we encountered an unweighted respondent size of less than 25, the confidence intervals were suppressed. Age-standardization of prevalence estimates was made for global comparison using the WHO World Population 2000-2025 [29]. Factors were checked for association with LBP by comparing LBP with no MSD through 2x2 tables. Univariate logistic regression analysis was done to obtain unadjusted odds ratios. All statistically significant relationships ($P<0.05$) were entered into a model for logistic regression analysis. The adjusted odds ratios and their 95% confidence limits were calculated to identify the strength of association of LBP factors. A detailed description of categorization and analysis of other variables was described elsewhere [19].

Ethics Approval and Consent to Participate:

Ethical guidelines, as outlined by the Declaration of Helsinki, were followed throughout the study [30]. Ethical clearance was obtained from the Institutional Review Board of

For peer review only

Bangabandhu Sheikh Mujib Medical University (ID 1100). Informed written consent was obtained from the respondents in Bangla as per Institutional Review Board's guidelines.

Patient and Public Involvement:

Patient or the public were not involved in the design, or conduct, or reporting, or dissemination plans of this study.

RESULTS

Characteristics of respondents:

In this nationally representative study, 2,000 adults 18 years or older were approached, and 1,843 (92.2%) agreed to participate [19]. The mean age of the participants was 40.5 (standard deviation 14.7) years, and 51.6% were women. A total of 561 (30.4%) had some type of MSDs. LBP was the most common among MSDs (18.6%, unadjusted), followed by knee osteoarthritis (7.3%) and soft-tissue rheumatism (5.2%). Among the inflammatory rheumatic diseases, the common conditions were rheumatoid arthritis (1.6%) and spondyloarthritis (1.3%).

Table 1 shows the changes brough in by the weighting procedure on the unweighted sample. The weighted percentages are more in line with the projected Population Frame [20] from which the study sample was drawn.

Characteristics of respondents with LBP:

Prevalence:

We report here (**Table 2**) weighted prevalence of LBP was 18.5% (95% CI: 11.8–25.2). However, the age-standardized prevalence of LBP to be 19.4% (95% CI: 14.0–24.8), which is significantly higher in women (27.2%, 19.3-35.1) than men (14.0%, 8.7–19.3). There has been a persistent increase in prevalence from 10.5% (95% CI 5.7–15.4) in 18–34 years age group to 27.8% (95% CI 16.1–39.5) in 55–99 years age group. This trend was more prominent in women. The prevalence did not vary significantly among occupational groups. People with no formal education had significantly highest prevalence of LBP (31.3%, 22.3–40.4) compared to other educational groups. Although the highest prevalence (23.5% (13.9–33.0)) was observed in the 1st quartile of the wealth index, it did not vary significantly. LBP

For peer review only - http://bmjopen.bmj.com/site/about/guidelines.xhtml

was not significantly associated with strenuous physical activity in our sample. We checked LBP prevalence by urban (14.6%, 9.9–19.2) and rural (20.2%, 11.0.6–29.3) categories, but it did not differ significantly. Among the co-morbidities, the prevalence of LBP was higher among patients of hypertension (26.7%, 15.0–38.4) and obesity (20.6%, 13.0–28.3). The highest prevalence of LBP (87.3%, 80.2–94.4) was seen in respondents who had multiple (two or more) MSDs such as LBP, knee osteoarthritis, soft tissue rheumatism, non-inflammatory MSDs, cervical spondylosis etc. **(Figure 2)**

Background characteristics:

Among the LBP respondents (n=343), 63.3% were women, and 65.3% were from rural areas. Mean age in years (95% confidence interval) was 45.3 (43.0–47.7) overall, and 48.3 (45.8–50.9) in men and 44.0 (41.0–47.0) in women. The study participants were divided into three subgroups as per age, and the highest number of LBP was observed in the 35–54 age group. More than half (%, 95% confidence interval: 57.4%, 48.2–66.6) were homemakers (all women), while the rest constituted other occupations like laborer, business professional, service holder and others. Almost half of the participants with LBP had no formal education (53.2%, 41.6–64.9). Overall, according to the wealth index, 33.2% (22.6–43.9) of respondents belonged to the 1st quartile (lowest socioeconomic status). About three-fourth of the respondents (77%, 55.9–98.0) had rural residence. **(Table 3)**

Disability and work loss:

The distribution of Bengali HAQ-DI scores was not normally distributed among the LBP patients. The scores ranged from 0–2.6. The LBP group's median (interquartile range) was 0.6 (0.3–0.9). The difference of Bangla HAQ-DI score between LBP (n=343) and no MSD (n=60) is statistically significant (P<0.0001) by the Kruskal-Wallis H test, indicating that LBP is associated with a higher disability. The distribution of days lost from work for LBP group had a highly skewed distribution and ranged from 0–365 days. However, the difference of days lost from work (n=1,625) between LBP (n=343) and no MSD (n=1,282) is statistically significant (P<0.0001) by the Kruskal-Wallis H test, indicating that LBP is associated with more days lost.

Factors associated:

For peer review only - http://bmjopen.bmj.com/site/about/guidelines.xhtml

Univariate logistic regression analysis did not show any significant association of LBP with occupation, strenuous physical activity, wealth indices, tobacco use, obesity, and diabetes (**Table 4**). A significant association was observed for age group 35–99 years, female sex, lack of education, history of physical trauma, and hypertension according to the unadjusted odds ratio and their 95% confidence intervals (P<0.05). These significant associations (*P*<0.01) persisted in the multiple logistic regression analysis having age sex, education and hypertension. into the model simultaneously.

DISCUSSION

LBP is a common medical problem with very high personal and societal impact, leading to poor quality of life and workability [31]. In this survey we report that one in five adults in Bangladesh suffers from LBP. The prevalence in Bangladesh is similar to Kuwait (22.7%) [32] and Qatar (23.3%) [33] but lower than northern India (32%) [34] and Iran (29.3%) [14]. Malaysia (11.6%) [35] has a lower prevalence of LBP than we report here. In some previous studies in Bangladesh the prevalence was found 25.6% among medical students [36], 36.6% in bank employees [37], 60.8% among physiotherapists [38]. Lack of maintenance of correct posture during sitting and scarcity of knowledge, understanding, or application of ergonomics are responsible for high prevalence rate among these groups [36, 38].

A systematic review of 165 studies from 54 countries revealed the global prevalence of LBP of 12-33% [1]. According to the systematic analysis of the Global Burden of Disease Study 2017, LBP was the leading cause of YLDs (years lived with disability) [39]. In our study, the rural people had a higher prevalence of LBP than the urban people. This difference–though not statistically significant–was probably due to lower doctor concentration in the rural areas, financial limitations, and less education status. However, regional variation was observed in other studies [14].

Age was a factor associated with LBP in this study. The prevalence of LBP persistently increased with age but this was not statistically significant. However, an analysis reported that the risk and prevalence of low back pain increased with age [5]. A systematic review of the global prevalence of LBP revealed the association of age was highest in the 40-49 age group [1, 40]. The overall prevalence rises with age 65, which gradually reduces thereafter

For peer review only

[41]. Some possible explanations are LBP characteristics in older adults that differ from the middle-aged population (less intense back pain, more leg pain, and more depression) [42]. Our study showed a higher prevalence of LBP among women than men, consistent with some other analyses [1, 41]. This could be due to more household or domestic activities among women compared to men. This finding agrees with the results from the national health survey on the Iranian population [14]. Another Indian study found no significant difference in age and sex scores in their study [43]. Higher women prevalence can be partially explained that they have a lower pain threshold than men [44]. The sex differences may be implied with gonadal steroid hormones like estradiol and testosterone that modulate sensitivity to pain and analgesia [45]. Women always experienced a higher frequency of visceral pain (e.g., during menstruation, pregnancy) than men [46]. It seems that in painful conditions, women exhibit a greater prevalence than men as women report more pain [47].

Leboeuf-Yde considered body weight as a possible weak indicator for LBP in his systematic literature review due to lack of evidence [48]. The results obtained in our study did not demonstrate a statistically significant association between LBP and higher BMI. Some other studies found obesity or high BMI associated with increased risk LBP development and severity [14, 49]. However, a cross-sectional study including nine countries found BMI $\geq$25kg/m^2 as a risk factor for LBP in five countries (Finland, Poland, Russia, South Africa and Spain), whereas it was not associated with LBP in the remaining four countries (China, Ghana, India and Mexico) [50].

In our research, we found that the absence of formal education is significantly associated with LBP. A cross-sectional study in the United States found that LBP is more common in people who have had less than high school education [51]. Other studies in the USA, UK, and Iran found lower educational status had an increased association with LBP and found higher education inversely associated with LBP [14, 52]. Several proposed mechanisms may account for the relationship between low academic status and back pain. The amount of formal education contributes to the types of jobs that an individual may involve in, and subsequently, the types of jobs influence LBP [53]. Moreover, health education regarding posture management, lifestyle changes, physical exercises, stress management poorly reached among people with an absence of formal education. We didn't find any significant association

For peer review only

For peer review only - http://bmjopen.bmj.com/site/about/guidelines.xhtml

of LBP with occupation. However, in our opinion, the larger number of homemakers affected with LBP might be linked with their nature of heavy work such as squatting, bending lifting heavy objects, prolonged standing etc. in the household. In a US-based study, LBP was significantly related to occupational factors such as truck driving, lifting, carrying, pulling, pushing, twisting, and non-driving vibrational exposure [54]. In some European countries, workers involved in heavy weightlifting (≥25 kg) suffered more from LBP [55].

Studies reported associations between LBP and lower social class [14, 51] but we did not find any significant association of LBP with economic status. This finding is coherent with a Danish study where they did not find any possible relationship between socioeconomic status and LBP [56]. In our analysis, trauma tended to be associated (unadjusted OR) with overall LBP, but the association was lost after adjustment. Trauma is not supposed to lead to chronic LBP and the persistence of back pain was more associated with psychological factors like stress, low education status, etc., than trauma itself [57]. We found a positive relationship between hypertension and LBP. LBP may cause inactivity and lack of exercise resulting in weight gain, subsequently creating or exaggerating co-morbid conditions like hypertension and diabetes mellitus. The Hong Kong Disc Degeneration-Cardiovascular Cohort showed that HTN is increases the possibility of moderate or severe disc degeneration which is highly associated with LBP [58]. Another long-term Finish study revealed that both SBP and DBP were positively associated with LBP suggesting atherosclerosis of lumbar vessels a possible mechanism of development of LBP [59]. In a Korean survey, the lifetime prevalence of LBP was 34.4% among the hypertensive respondents, but the adjusted OR of LBP prevalence was significantly lower than the normotensive subjects (fully adjusted 0.79, 95% CI 0.70-0.90) [60]. A Bangladeshi study conducted among the doctors working in a tertiary care hospital found that HTN was the most common comorbid condition among the LBP sufferers [61]. There was no association between diabetes and LBP in this study.

Strengths and Limitations

This is the first nationally representative survey report on LBP in Bangladesh, and probably, among all south Asian nations. Although we have weighted the data for national representation, the sample size calculation for the original study was based on point prevalence of MSD [19]. We now know that the prevalence of LBP was 19.4% and

For peer review only

For peer review only - http://bmjopen.bmj.com/site/about/guidelines.xhtml

prevalence of MSD was 30.4%. A larger sample size maintaining adequate power was needed for generalizability of the study results. Therefore, a cautious interpretation is necessary because of inadequate sample size, especially when split into reporting domains. We have operationally defined the recall period for reporting work loss days as 12 months which might induce bias. Trained rheumatology residents diagnosed the patients which was verified by experienced rheumatologists in the field. Some diagnoses of evolving rheumatological conditions might lack sufficient validity because of a lack of adequate laboratory facilities in the field.

CONCLUSION

This nationally representative study reports the population weighted prevalence of LBP by sociodemographic background, comorbidities and associated factors in Bangladesh. One in five adults suffer from LBP. Education and hypertension are modifiable factors that warrant intervention. Increase in level of education, care to middle and older population, and good control of hypertension may reduce LBP burden. A special attention is needed to prevent LBP in women. Further study with a larger sample size addressing these neglected issues may have more clarifications to decrease the burden of LBP.

For peer review only

Abbreviations and acronyms:

BMI	Body mass index
BSMMU	Bangabandhu Sheikh Mujib Medical University
CI	Confidence interval
COPCORD	Community oriented program from control of rheumatic diseases
HAQ-DI	Health Assessment Questionnaire - Disability Index
LBP	Low back pain
MSK	Musculoskeletal
MSD	Musculoskeletal disorder
OR	Odds ratio
PPS	Population proportion to size
PSU	Primary sampling unit
WHO	World health organization

Acknowledgement: We gratefully acknowledge the field team, divisional coordinators, civil surgeons, upazila health and family planning officers and health assistants for their support.

Author contribution:

Muhammad Shoaib Momen Majumder (MSMM): Conceptualized the area of work, and prepared the first draft of the manuscript and revised the subsequent drafts.

Ferdous Hakim (FH): analyzed the data, guided preparation of graphs and tables, interpreted the results, revised the draft manuscript, and coordinated the writing exercise.

Iftekhar Hussain Bandhan (IHB): prepared graphs and tables, revised the draft manuscript.

Mohammad Abdur Razzaque (MAR): reviewed the manuscript and with special reference to the literature review.

Ahmed Zahid-Al-Quadir and Shamim Ahmed: Prepared the training manual, trained the field team, executed the field operation in coordination with the divisional investigators;

Minhaj R Choudhury, Syed Atiqul Haq and Mohammad Mostafa Zaman: Designed the study, guided the analysis and manuscript writing, critically reviewed the results and manuscript drafts, ensured integrity of data.

All authors: Approved publication.

For peer review only

For peer review only - http://bmjopen.bmj.com/site/about/guidelines.xhtml

Funding: Preparing this manuscript did not require any funding. However, the base study was supported by the World Health Organization, Bangladesh (Agreement Reference: SEBAN140895) back in 2015. As a part of its mandate to strengthen national research capacity, WHO provided technical guidance in designing, implementing, analysing data, and writing the report. However, it does not have any influence on the results.

Consent to publish: All authors contributed to the manuscript and provided consent to publish.

Availability of data and materials: The dataset used for this manuscript is available with the corresponding author upon reasonable request.

Disclosure of Conflicts of Interest: None of the authors has any conflict of interest to disclose. The authors alone are responsible for their views expressed in this article, which do not necessarily represent the institutions' views, decisions, or policies with which they are affiliated.

Ethical Publication Statement: We confirm that we have read the Journal's position on issues involved in ethical publication and affirm that this report is consistent with those guidelines.

For peer review only

Tables

Table 1. Characteristics of the respondents with LBP by unweighted and weighted numbers, Musculoskeletal Disease Survey 2015

Sociodemographic characteristics	Unweighted sample (n=1,843) number (percent)	Weighted* sample (N=94,794,164) number (percent)
Total	1843 (100.0)	94,794,164 (100.0)
Sex		
Men	892 (48.4)	41,553,976 (43.8)
Women	951 (51.6)	53,240,188 (56.2)
Residence		
Urban	716 (38.9)	27,772,657 (29.3)
Rural	1127 (61.2)	67,021,507 (70.7)
Age, years		
18-34	711 (38.6)	41,343,470 (43.6)
35-54	774 (42.0)	35,278,850 (37.2)
55-99	358 (19.4)	18,171,844 (19.2)

* Weighted to projected population of Bangladesh from 2001 Population Census Frame of Bangladesh Bureau of Statistics.

For peer review only - http://bmjopen.bmj.com/site/about/guidelines.xhtml

Table 2. Weighted prevalence of low back pain by sociodemographic characteristics in Bangladesh, Musculoskeletal Disease Survey 2015

Sociodemographic characteristics	Total	Men	Women
	Percent (95% CI)	Percent (95% CI)	
Overall	18.5 (11.8–25.2)	13.1 (6.4–19.9)	22.7 (15.3–30.2)
Overall (age-standardized)*	19.4 (14.0–24.8)	14.0 (8.7–19.3)	27.2 (19.3–35.1)
Age in years			
18-34	10.5 (5.7–15.4)	5.2 (1.3–9.0)	13.5 (7.2–19.9)
35-54	23.1 (15.3–30.9)	18.8 (8.7–28.8)	26.7 (18.3–35.1)
55-99	27.8 (16.1–39.5)	15.7 (6.7–24.7)	44.5 (23.9–65.1)
Occupation			
Homemaker	23.6 (15.9–31.2)	–	23.6 (15.9–31.2)
Laborer†	18.2 (9.4–27.0)	17.9 (8.6–27.3)	21.2 (6.2–36.3)
Business professional	9.6 (1.9–17.2)	9.8 (1.9–17.6)	–
Service holder	10.7 (1.2–20.1)	10.3 (0.4–20.2)	–
Others†	13.9 (4.1–23.6)	8.6 (1.7–15.5)	20.3 (6.0–34.6)
Education			
No formal education (0)	31.3 (22.3–40.4)	20.3 (13.4–27.2)	37.4 (24.3–50.5)
Any primary education (1-5)	13.3 (6.7–19.9)	12.1 (3.9–20.3)	14.4 (7.8–21.0)
Any secondary education (6-10)	14.9 (7.4–22.3)	12.0 (2.4–21.6)	17.5 (9.9–25.0)
Above secondary (≥11 years)	6.9 (3.8–10.1)	–	8.0 (3.7–12.3)
Married‡	19.6 (12.4–26.7)	14.1 (6.7–21.4)	23.7 (15.7–31.7)
Wealth index quartile§			
1st	23.5 (13.9–33.0)	19.5 (11.2–27.8)	25.5 (12.9–38.2)
2nd	19.8 (8.4–31.2)	17.3 (3.7–30.9)	21.6 (10.3–32.9)
3rd	14.1 (7.6–20.5)	8.3 (2.3–14.2)	19.8 (11.7–28.0)
4th	16.6 (9.3–23.8)	10.3 (3.2–17.4)	23.1 (11.5–34.7)
Residence			
Urban	14.6 (9.1–20.0)	9.8 (3.9–15.6)	17.7 (8.5–26.8)
Rural	20.2 (10.5–29.8)	14.3 (4.8–23.9)	25.1 (14.7–35.4)
Strenuous physical activity‖	17.1 (4.3–29.8)	17.2 (4.0–30.4)	–
History of physical trauma	24.5 (14.2–34.7)	24.6 (13.0–36.2)	24.4 (7.9–40.9)

All values are percent (95% confidence interval). Weighted percentages shown are calculated from Census 2001 Population Frame by Bangladesh Bureau of Statistics to reflect projected population of Bangladesh (N=94,794,164).

Bold face values denote statistically significant higher prevalence in women compared to men.

* Standardized for WHO World Population 2000-2020.

† Labourer includes: farmer, daily worker, rickshaw puller, garments worker, field worker and others. Other occupations: retired, weaver and housekeeper

‡ Includes currently married, divorced, separated and widowed.

§ The wealth index was constructed using principal component analysis out of a list of 20 household assets (see Methods section for details);

‖ Fourth quartile of the MET-minutes distribution of work-related physical activity. Commutation and leisure time physical activities were not considered because these were negligible contributors.

– 95% confidence interval not reported as number of respondents are <25.

For peer review only - http://bmjopen.bmj.com/site/about/guidelines.xhtml

Table 3. Socioeconomic characteristics of patients of low back pain in Bangladesh, Musculoskeletal Disease Survey 2015

**Sociodemographic characteristics**	**Total**	**Men**	**Women**
	**Weighted percentage (95% confidence interval)**		
Age in years			
Mean (95% confidence interval)	45.3 (43.0–47.7)	48.3 (45.8–50.9)	44.0 (41.0–47.0)
18–34	24.8 (18.8–30.7)	14.1 (6.9–21.3)	29.6 (21.3–37.9)
35–54	46.4 (40.6–52.2)	55.6 (40.3–70.9)	42.3 (34.4–50.2)
55–99	28.8 (22.5–35.1)	30.3 (17.0–43.7)	28.1 (22.2–34.0)
Occupation			
Homemaker	57.4 (48.2–66.6)	–	83.2 (72.0–94.5)
Laborer†	20.4 (13.6–27.2)	59.7 (38.8–80.5)	2.7 (0.1–5.4)
Business professional	5.2 (1.2–9.1)	16.6 (5.0–28.2)	–
Service holder	–	–	–
Others†	13.5 (2.7–24.3)	14.8 (2.8–26.8)	13.0 (1.5–24.4)
Education			
No formal education (0)	53.2 (41.6–64.9)	39.1 (24.3–54.0)	59.6 (46.9–72.3)
Any primary education (1-5)	17.2 (13.8–20.5)	23.9 (17.1–42.6)	14.1 (10.1–18.1)
Any secondary education (6-10)	24.1 (15.9–32.4)	29.9 (17.1–42.6)	21.6 (13.1–30.1)
Above secondary (≥11 years)	5.5 (1.0–9.9)	–	–
Married‡	97.8 (94.8–100.8)	96.8 (92.2–101.3)	98.3 (95.5–101.1)
Wealth index quartile§			
1st	33.2 (22.6–43.9)	30.1 (18.8–41.4)	34.7 (21.7–47.7)
2nd	25.5 (14.8–36.2)	29.5 (11.5–47.5)	23.7 (14.6–32.7)
3rd	19.7 (11.0–28.4)	18.5 (6.7–30.3)	20.3 (10.5–30.1)
4th	21.6 (9.2–33.9)	21.9 (9.4–34.5)	21.4 (7.1–35.7)
Rural residence	77.0 (55.9–98.0)	80.3 (60.2–100.4)	75.5 (52.8–98.1)

* All values are percent (95% confidence interval) unless stated otherwise. Weighted percentages shown are calculated from Census 2001 Population Frame by Bangladesh Bureau of Statistics to reflect projected population of Bangladesh (N=94,794,164).

† Labourer include: farmer, daily worker, rickshaw puller, garments worker, field worker and others. Other occupations: retired, weaver and housekeeper etc.

‡ Includes currently married, divorced, separated and widowed.

§ The wealth index was constructed using principal component analysis out of a list of 20 household assets (see Methods section for details);

\- numbers are low

For peer review only

For peer review only - http://bmjopen.bmj.com/site/about/guidelines.xhtml

Table 4: Odds ratios of factors associated with low back pain compared with no musculoskeletal disorders in Bangladeshi adults, Musculoskeletal Disease Survey 2015

Factors	Odds ratio (95% confidence interval)	
	**Unadjusted**	**Adjusted**
Age group, years	3.2 (2.5–4.2)**	2.4 (1.7–3.4)**
(35–99=1, 18–34=0)	1.0	1.0
Sex	2.1 (1.3–3.3)**	2.2 (1.5–3.3)**
(woman=1, man=0)	1.0	1.0
Labourer†	0.9 (0.7–1.3)	-
(yes=1, no=0)	1.0	-
No formal education	3.5 (2.5–5.0)**	2.3 (1.6–3.3)**
(yes=1, no=0)	1.0	1.0
Low wealth index	1.6 (1.1–2.3)**	1.0 (0.6–1.5)
(yes=1, no=0)	1.0	1.0
Strenuous physical activity‡	0.8 (0.4–1.6)	-
(yes=1, no=0)	1.0	-
Obesity (body mass index ≥25 Kg/m^2)	1.3 (0.8–2.1)	-
(yes=1, no=0)	1.0	-
History of physical trauma	1.8 (1.1–3.2)*	1.6 (0.9–2.8)
(yes=1, no=0)	1.0	1.0
Current tobacco user	1.1 (0.8–1.6)	-
(yes=1, no=0)	1.0	-
Hypertension	2.3 (1.3–4.0)**	1.7 (1.1–2.6)*
(yes=1, no=0)	1.0	1.0
Diabetes mellitus	1.0 (0.5–1.7)	-
(yes=1, no=0)	1.0	-

LBP: low back pain; MSD: musculoskeletal disorder
* $P<0.05$, ** $P<0.01$

† Labourer includes: farmer, daily worker, rickshaw puller, garments worker, field worker and others.
‡ Fourth quartile of the MET-minutes distribution of work-related physical activity. Commutation and leisure time physical activities were not considered because these were negligible contributors.

For peer review only

For peer review only - http://bmjopen.bmj.com/site/about/guidelines.xhtml

455 **Figures**

456 **Fig. 1 Flow chart on the selection of low back pain patients from the national survey on musculoskeletal conditions in Bangladesh (2015) adapted from Ahmad Zahid-Al-Quadir *et al* [21]**

460 * Eight divisions from Sept 2015. Randomly 15 districts were selected out of 64.
461 ** PPS indicates population proportion to size.
462 *** Out-migration, broken house, locked house, no tenant, out of home, refusal. Two recall visits were done if the selected house was locked and the person chosen was not available at home at the time of the interviewer's visit. In case of non-participation after the second recall visit, the targeted household/individual was declared non-respondents.

For peer review only

For peer review only - http://bmjopen.bmj.com/site/about/guidelines.xhtml

Fig. 2 Weighted prevalences (percent) of low back pain in co-morbid conditions (error bars indicate 95% confidence intervals)

*Diabetes was defined as random capillary glucose level ≥11.1 or on medication for diabetes

†Obesity is defined as, body mass index ≥25 kg/m^2;

‡Hypertension was defined as systolic blood pressure ≥140 or diastolic blood pressure ≥90 or medication for hypertension;

For peer review only

References

1. Hoy D, Bain C, Williams G, et al. A systematic review of the global prevalence of low back pain. Arthritis Rheum. 2012 Jun;64(6):2028-37. doi: 10.1002/art.34347.
2. Dionne CE, Dunn KM, Croft PR, et al. A consensus approach toward the standardization of back pain definitions for use in prevalence studies. Spine (Phila Pa 1976). 2008 Jan 1;33(1):95-103. doi: 10.1097/BRS.0b013e31815e7f94.
3. Deyo RA, Tsui-Wu YJ. Descriptive epidemiology of low-back pain and its related medical care in the United States. Spine (Phila Pa 1976). 1987 Apr;12(3):264-8. doi: 10.1097/00007632-198704000-00013.
4. Balagué F, Mannion AF, Pellisé F, et al. Non-specific low back pain. Lancet. 2012 Feb 4;379(9814):482-91. doi: 10.1016/S0140-6736(11)60610-7.
5. Andersson GB. Epidemiological features of chronic low-back pain. Lancet. 1999 Aug 14;354(9178):581-5. doi: 10.1016/S0140-6736(99)01312-4.
6. Fatoye F, Gebrye T, Odeyemi I. Real-world incidence and prevalence of low back pain using routinely collected data. Rheumatol Int. 2019 Apr;39(4):619-626. doi: 10.1007/s00296-019-04273-0.
7. Hartvigsen J, Hancock MJ, Kongsted A, et al. Lancet Low Back Pain Series Working Group. What low back pain is and why we need to pay attention. Lancet. 2018 Jun 9;391(10137):2356-2367. doi: 10.1016/S0140-6736(18)30480-X.
8. Parreira P, Maher CG, Steffens D, et al. Risk factors for low back pain and sciatica: an umbrella review. Spine J. 2018 Sep;18(9):1715-1721. doi: 10.1016/j.spinee.2018.05.018.

For peer review only

9. Lötters F, Burdorf A. Prognostic factors for duration of sickness absence due to musculoskeletal disorders. The Clinical Journal of Pain. 2006 Feb;22(2):212-221. DOI: 10.1097/01.ajp.0000154047.30155.72.

10. Guo HR, Tanaka S, Halperin WE, et al. Back pain prevalence in US industry and estimates of lost workdays. Am J Public Health. 1999 Jul;89(7):1029-35. doi: 10.2105/ajph.89.7.1029.

11. Cassidy JD, Carroll LJ, Côté P. The Saskatchewan health and back pain survey. The prevalence of low back pain and related disability in Saskatchewan adults. Spine. 1998 Sep 1;23(17):1860-6; discussion 1867. doi: 10.1097/00007632-199809010-00012.

12. Harreby M, Kjer J, Hesselsøe G, Neergaard K. Epidemiological aspects and risk factors for low back pain in 38-year-old men and women: a 25-year prospective cohort study of 640 school children. Eur Spine J. 1996;5(5):312-8. doi: 10.1007/BF00304346.

13. Waxman R, Tennant A, Helliwell P. A prospective follow-up study of low back pain in the community. Spine. 2000 Aug 15;25(16):2085-90. doi: 10.1097/00007632-200008150-00013.

14. Biglarian A, Seifi B, Bakhshi E, et al. Low back pain prevalence and associated factors in Iranian population: findings from the national health survey. Pain Res Treat. 2012;2012:653060. doi: 10.1155/2012/653060.

15. Gupta G, Nandini N. Prevalence of low back pain in non working rural housewives of Kanpur, India. Int J Occup Med Environ Health. 2015;28(2):313-20. doi: 10.13075/ijomeh.1896.00299.

For peer review only - http://bmjopen.bmj.com/site/about/guidelines.xhtml

16. Ahdhi GS, Subramanian R, Saya GK, et al. Prevalence of low back pain and its relation to quality of life and disability among women in rural areas of Puducherry, India. Indian J Pain [serial online] 2016 [cited 2021 Apr 18];30:111-5. Available from: https://www.indianjpain.org/text.asp?2016/30/2/111/186467.

17. Bansal D, Asrar MM, Ghai B, et al. Prevalence and Impact of Low Back Pain in a Community-Based Population in Northern India. Pain Physician. 2020;23(4):E389-E98.

18. Haq SA, Darmawan J, Islam MN, et al. Prevalence of rheumatic diseases and associated outcomes in rural and urban communities in Bangladesh: a COPCORD study. J Rheumatol. 2005 Feb;32(2):348-53. PMID: 15693098.

19. Zahid-Al-Quadir, A., Zaman, M.M., Ahmed, S. et al. Prevalence of musculoskeletal conditions and related disabilities in Bangladeshi adults: a cross-sectional national survey. BMC Rheumatol 4, 69 (2020). https://doi.org/10.1186/s41927-020-00169-w

20. Bangladesh Bureau of Statistics. Population and housing census 2001, preliminary result. Dhaka: Bangladesh Bureau of Statistics; 2001. Accessed 20 Apr 2020. http://bbs.portal.gov.bd.

21. Islam, ME, Ahmed, S, Shahin, MA, et al. Cross-cultural adaptation and validation of revised WHO-ILAR-COPCORD core English questionnaire translated to Bangla. Int J Rheum Dis. 2021; 00: 1– 14. https://doi.org/10.1111/1756-185X.14176

22. Beaton DE, Bombardier C, Guillemin F, Ferraz MB. Guidelines for the process of cross-cultural adaptation of self-report measures. Spine (Phila Pa 1976). 2000 Dec 15;25(24):3186-91. doi: 10.1097/00007632-200012150-00014.

23. Chassany O, Marquis P, Scherrer B, Read NW, Finger T, Bergmann JF, Fraitag B, Geneve J, Caulin C. Validation of a specific quality of life questionnaire for

For peer review only - http://bmjopen.bmj.com/site/about/guidelines.xhtml

functional digestive disorders. Gut. 1999 Apr;44(4):527-33. doi: 10.1136/gut.44.4.527.

24. van Tulder M, Becker A, Bekkering T, et al. COST B13 Working Group on Guidelines for the Management of Acute Low Back Pain in Primary Care. Chapter 3. European guidelines for the management of acute nonspecific low back pain in primary care. Eur Spine J. 2006 Mar;15 Suppl 2(Suppl 2):S169-91. doi: 10.1007/s00586-006-1071-2.

25. Zaman MM, Rahman MM, Rahman MR, et al. Prevalence of risk factors for non-communicable diseases in Bangladesh: Results from STEPS survey 2010. Indian J Public Health. 2016 Jan-Mar;60(1):17-25. doi: 10.4103/0019-557X.177290.

26. Islam N, Baron Basak T, OudeVoshaar MA, et al. Cross-cultural adaptation and validation of a Bengali Health Assessment Questionnaire for use in rheumatoid arthritis patients. Int J Rheum Dis. 2013 Aug;16(4):413-7. doi: 10.1111/1756-185X.12032.

27. Misra A. Ethnic-Specific Criteria for Classification of Body Mass Index: A Perspective for Asian Indians and American Diabetes Association Position Statement. Diabetes Technol Ther. 2015 Sep;17(9):667-71. doi: 10.1089/dia.2015.0007.

28. Hakim, F., Bhuiyan, R., Akter, M. K., Mohit, M. A., Alam, M. F., Karim, M. R., & Zaman, M. M. (2022). Weighting National Survey Data in Bangladesh: Why, How and Which weight? Weighting survey data. Bangladesh Medical Research Council Bulletin, 47(2), 118–126. https://doi.org/10.3329/bmrcb.v47i2.57769

29. Ahmad OB, Boschi-Pinto C, Lopez AD, et al. Age Standardization of Rates: A New WHO Standard. GPE Discussion Paper Series: No31 Geneva, Switzerland: World Health Organization, 2001.

For peer review only - http://bmjopen.bmj.com/site/about/guidelines.xhtml

30. WMA Declaration of Helsinki - ethical principles for medical research involving human respondents. [Cited on: 2020 Aug 29] Available from: https://www.wma.net/policies-post/wma-declaration-of-helsinki-ethical-principlesfor-medical-research-involving-human-respondents/.

31. Dutmer AL, Schiphorst Preuper HR, Soer R, et al. Personal and Societal Impact of Low Back Pain: The Groningen Spine Cohort. Spine (Phila Pa 1976). 2019 Dec 15;44(24):E1443-E1451. doi: 10.1097/BRS.0000000000003174.

32. Al-Awadhi AM, Olusi SO, Al-Saeid K, et al. Incidence of musculoskeletal pain in adult Kuwaitis using the validated Arabic version of the WHO-ILAR COPCORD Core Questionnaire. Annals of Saudi Medicine. 2005 Nov-Dec;25(6):459-462. DOI: 10.5144/0256-4947.2005.459.

33. Aldeen Sarakbi H, Alsaed O, Hammoudeh M, et al.. Epidemiology of musculoskeletal complaints and diseases in Qatar: A cross-sectional study. Qatar Med J. 2020 Nov 12;2020(2):29. doi: 10.5339/qmj.2020.29.

34. Bansal D, Asrar MM, Ghai B, et al. Prevalence and Impact of Low Back Pain in a Community-Based Population in Northern India. Pain Physician. 2020 Jul;23(4):E389-E398. PMID: 32709185.

35. Veerapen K, Wigley RD, Valkenburg H. Musculoskeletal pain in Malaysia: a COPCORD survey. J Rheumatol. 2007 Jan;34(1):207-13. PMID: 17216688.

36. Sany SA, Tanjim T and Hossain MI. Low back pain and associated risk factors among medical students in Bangladesh: a cross-sectional study. F1000Research. 2021 July; 10:698. Doi: 10.12688/f1000research.55151.1.

For peer review only
For peer review only - http://bmjopen.bmj.com/site/about/guidelines.xhtml

37. Ali M, Ahsan GU, Hossain A. Prevalence and associated occupational factors of low back pain among the bank employees in Dhaka City. J Occup Health. 2020 Jan; 62(1):e12131. doi: 10.1002/1348-9585.12131.

38. Mondal R, Sarker RC, Akter S, Banik PC, Baroi SK. Prevalence of low back pain and its associated factors among physiotherapists in Dhaka city of Bangladesh in 2016. Journal of Occupational Health and Epidemiology. 2018. 7:70–4. Doi: 10.29252/johe.7.2.70.

39. GBD 2017 Disease and Injury Incidence and Prevalence Collaborators. Global, regional, and national incidence, prevalence, and years lived with disability for 354 diseases and injuries for 195 countries and territories, 1990-2017: a systematic analysis for the Global Burden of Disease Study 2017. Lancet. 2018 Nov 10;392(10159):1789-1858. doi: 10.1016/S0140-6736(18)32279-7.

40. Hoy D, Brooks P, Blyth F, et al. The Epidemiology of low back pain. Best Practice & Research Clinical Rheumatology. 2010 Dec;24(6):769–81. doi: 10.1016/j.berh.2010.10.002.

41. Loney PL, Stratford PW. The prevalence of low back pain in adults: a methodological review of the literature. Phys Ther. 1999 Apr;79(4):384-96. PMID: 10201544.

42. Manogharan S, Kongsted A, Ferreira ML, et al. Do older adults with chronic low back pain differ from younger adults in regards to baseline characteristics and prognosis? Eur J Pain (United Kingdom). 2017;21(5):866–73.

43. Ganesan S, Acharya AS, Chauhan R, Acharya S. Prevalence and Risk Factors for Low Back Pain in 1,355 Young Adults: A Cross-Sectional Study. Asian Spine J. 2017 Aug;11(4):610-617. doi: 10.4184/asj.2017.11.4.610.

For peer review only - http://bmjopen.bmj.com/site/about/guidelines.xhtml

44. Yue P, Liu F, Li L. Neck/shoulder pain and low back pain among school teachers in China, prevalence and risk factors. BMC Public Health. 2012 Sep 14;12(1). doi: 10.1186/1471-2458-12-789.

45. Craft RM, Mogil JS, Aloisi AM. Sex differences in pain and analgesia: the role of gonadal hormones. European Journal of Pain. 2004 Oct;8(5):397–411. doi: 10.1016/j.ejpain.2004.01.003

46. Berkley KJ. Sex differences in pain. Behav Brain Sci. 1997 Sep;20(3):371–80. doi: 10.1017/s0140525x97221485.

47. Keogh E. Sex Differences in Pain. Reviews in Pain. 2008 Dec;2(2):4–7. doi: 10.1177/204946370800200203.

48. Leboeuf-Yde C. Body Weight and Low Back Pain. Spine. 2000 Jan;25(2):226. doi: 10.1097/00007632-200001150-00015.

49. Webb R, Brammah T, Lunt M, et al. Prevalence and Predictors of Intense, Chronic, and Disabling Neck and Back Pain in the UK General Population. Spine. 2003 Jun;28(11):1195–202. doi: 10.1097/01.BRS.0000067430.49169.01.

50. Koyanagi A, Stickley A, Garin N, Miret M, Ayuso-Mateos JL, Leonardi M, Koskinen S, Galas A, Haro JM. The association between obesity and back pain in nine countries: a cross-sectional study. BMC Public Health. 2015 Feb 11;15:123. doi: 10.1186/s12889-015-1362-9.

51. Shmagel A, Foley R, Ibrahim H. Epidemiology of Chronic Low Back Pain in US Adults: Data From the 2009-2010 National Health and Nutrition Examination Survey. Arthritis Care & Research. 2016 Oct 26;68(11):1688–94. doi: 10.1002/acr.22890.

For peer review only

For peer review only - http://bmjopen.bmj.com/site/about/guidelines.xhtml

52. Deyo RA, Tsui-wu YJ. Descriptive Epidemiology of Low-back Pain and Its Related Medical Care in the United States. Spine. 1987 Apr;12(3):264–8. doi: 10.1097/00007632-198704000-00013.

53. Rubin DI. Epidemiology and Risk Factors for Spine Pain. Neurologic Clinics. 2007 May;25(2):353–71. doi: 10.1016/j.ncl.2007.01.004.

54. Frymoyer JW, Pope MH, Costanza MC, et al. Epidemiologic Studies of Low-Back Pain. Spine. 1980 Sep;5(5):419–23. doi: 10.1097/00007632-198009000-00005.

55. Coggon D, Ntani G, Palmer KT, et al. Drivers of international variation in prevalence of disabling low back pain: Findings from the Cultural and Psychosocial Influences on Disability study. European journal of pain. 2018 Jun 27;23(1):35–45. doi: 10.1002/ejp.1255.

56. Hestbaek L, Korsholm L, Leboeuf-Yde C, Kyvik KO. Does socioeconomic status in adolescence predict low back pain in adulthood? A repeated cross-sectional study of 4,771 Danish adolescents. Eur Spine J. 2008 Dec;17(12):1727-34. doi: 10.1007/s00586-008-0796-5.

57. Harris IA, Young JM, Rae H, et al. Factors associated with back pain after physical injury: a survey of consecutive major trauma patients. Spine (Phila Pa 1976). 2007 Jun 15;32(14):1561-5. doi: 10.1097/BRS.0b013e318067dce8.

58. Samartzis D, Bow C, Karppinen J, Luk KDK, Cheung BMY, Cheung KMC. Hypertension is Independently Associated with Lumbar Disc Degeneration: A Large-Scale Population-Based Study. Global Spine Journal, vol. 4, no. 1_suppl, May 2014, p. s-0034-1376579-s-0034-1376579. doi:10.1055/s-0034-1376579.

For peer review only

For peer review only - http://bmjopen.bmj.com/site/about/guidelines.xhtml

707 59. Leino-Arjas P, Solovieva S, Kirjonen J, Reunanen A, Riihimäki H. Cardiovascular risk factors and low-back pain in a long-term follow-up of industrial employees. Scand J Work Environ Health. 2006 Feb;32(1):12-9. doi: 10.5271/sjweh.971.

710 60. Bae Y-H, Shin J-S, Lee J, et al. Association between Hypertension and the Prevalence of Low Back Pain and Osteoarthritis in Koreans: A Cross-Sectional Study. Fuchs FD, editor. PLoS ONE. 2015 Sep 22;10(9):e0138790. doi: 10.1371/journal.pone.0138790.

713 61. Rahman MA, Khan MA, Akter MR, Islam MA, Khan MK. Proportion of Low Back Pain among Physicians Working in a Tertiary Level Hospital of Bangladesh. Mymensingh Medical Journal: MMJ. 2021 Apr 1;30(2):262-6. PMID: 33830101

For peer review only

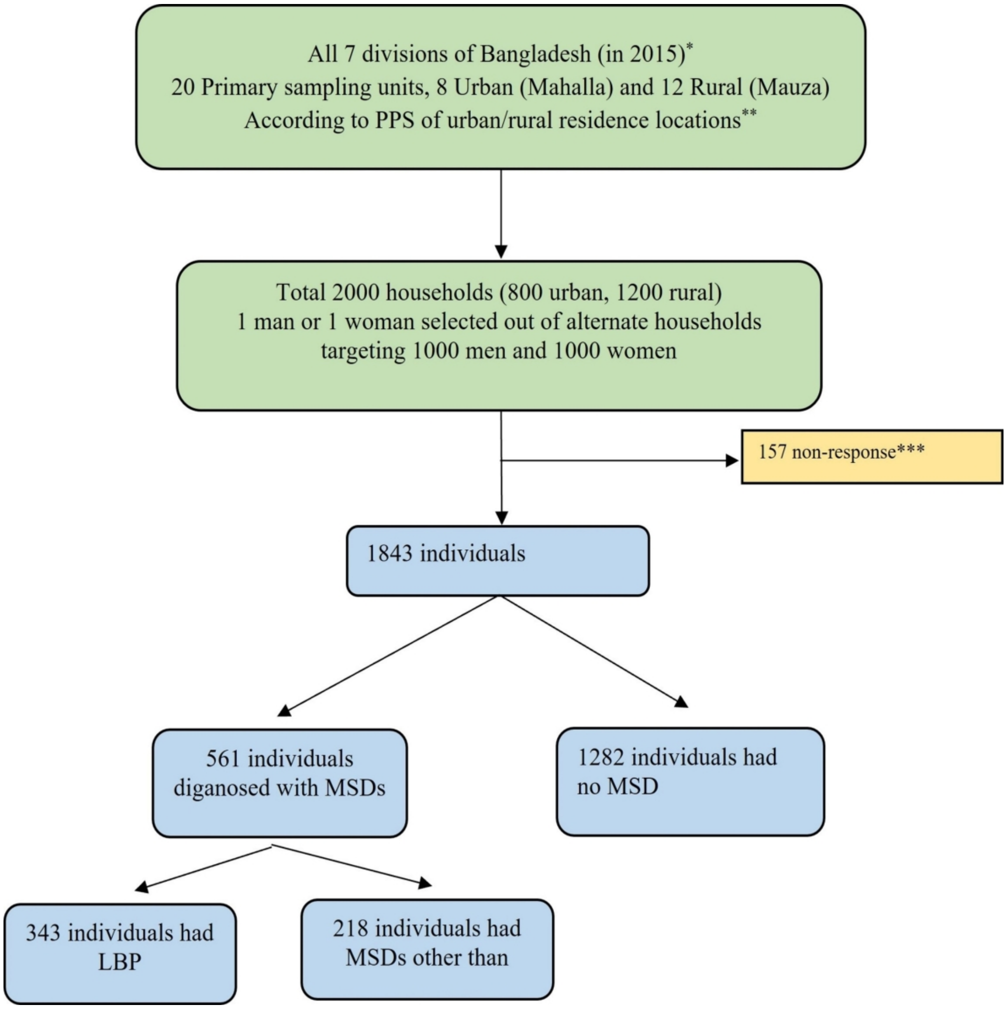

Caption:
Fig. 1 Flow chart on the selection of low back pain patients from the national survey on musculoskeletal conditions in Bangladesh (2015) adapted from Ahmad Zahid-Al-Quadir et al 21
Legend:
* Eight divisions from Sept 2015. Randomly 15 districts were selected out of 64.
** PPS indicates population proportion to size.
*** Out-migration, broken house, locked house, no tenant, out of home, refusal. Two recall visits were done if the selected house was locked and the person chosen was not available at home at the time of the interviewer's visit. In case of non-participation after the second recall visit, the targeted household/individual was declared non-respondents.

190x190mm (330 x 330 DPI)

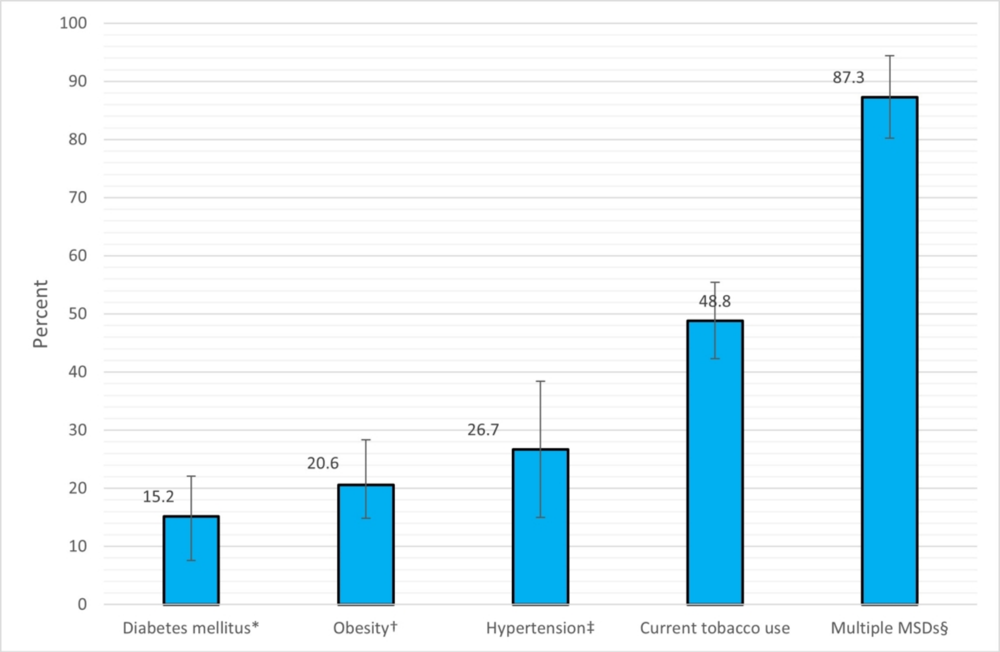

Caption:Fig. Weighted prevalences (percent) of low back pain in co-morbid conditions (error bars indicate 95% confidence intervals)Legend:*Diabetes was defined as random capillary glucose level ≥11.1 or on medication for diabetes†Obesity is defined as body mass index ≥25 kg/m2;‡Hypertension was defined as systolic blood pressure ≥140 or diastolic blood pressure ≥90 or medication for hypertension

597x389mm (330 x 330 DPI)

STROBE 2007 (v4) Statement—Checklist of items that should be included in reports of *cross-sectional studies*

Section/Topic	Item #	Recommendation	Reported on page #
**Title and abstract**	1	(*a*) Indicate the study's design with a commonly used term in the title or the abstract	1
		(*b*) Provide in the abstract an informative and balanced summary of what was done and what was found	2
**Introduction**			
Background/rationale	2	Explain the scientific background and rationale for the investigation being reported	4, 5
Objectives	3	State specific objectives, including any prespecified hypotheses	5
**Methods**			
Study design	4	Present key elements of study design early in the paper	5
Setting	5	Describe the setting, locations, and relevant dates, including periods of recruitment, exposure, follow-up, and data collection	5, 6
Participants	6	(*a*) Give the eligibility criteria, and the sources and methods of selection of participants	5
Variables	7	Clearly define all outcomes, exposures, predictors, potential confounders, and effect modifiers. Give diagnostic criteria, if applicable	6
Data sources/ measurement	8*	For each variable of interest, give sources of data and details of methods of assessment (measurement). Describe comparability of assessment methods if there is more than one group	5, 6
Bias	9	Describe any efforts to address potential sources of bias	5, 6
Study size	10	Explain how the study size was arrived at	5
Quantitative variables	11	Explain how quantitative variables were handled in the analyses. If applicable, describe which groupings were chosen and why	6
Statistical methods	12	(*a*) Describe all statistical methods, including those used to control for confounding	7
		(*b*) Describe any methods used to examine subgroups and interactions	7
		(*c*) Explain how missing data were addressed	7
		(*d*) If applicable, describe analytical methods taking account of sampling strategy	7
		(*e*) Describe any sensitivity analyses	Not applicable
**Results**			

For peer review only

Participants	13*	(a) Report numbers of individuals at each stage of study—eg numbers potentially eligible, examined for eligibility, confirmed eligible, included in the study, completing follow-up, and analysed	5, 22
		(b) Give reasons for non-participation at each stage	19
		(c) Consider use of a flow diagram	19
Descriptive data	14*	(a) Give characteristics of study participants (eg demographic, clinical, social) and information on exposures and potential confounders	7, 8
		(b) Indicate number of participants with missing data for each variable of interest	Not applicable
Outcome data	15*	Report numbers of outcome events or summary measures	8, 9
Main results	16	(*a*) Give unadjusted estimates and, if applicable, confounder-adjusted estimates and their precision (eg, 95% confidence interval). Make clear which confounders were adjusted for and why they were included	9
		(*b*) Report category boundaries when continuous variables were categorized	6
		(*c*) If relevant, consider translating estimates of relative risk into absolute risk for a meaningful time period	Not applicable
Other analyses	17	Report other analyses done—eg analyses of subgroups and interactions, and sensitivity analyses	Not applicable
**Discussion**			
Key results	18	Summarise key results with reference to study objectives	10
Limitations	19	Discuss limitations of the study, taking into account sources of potential bias or imprecision. Discuss both direction and magnitude of any potential bias	3
Interpretation	20	Give a cautious overall interpretation of results considering objectives, limitations, multiplicity of analyses, results from similar studies, and other relevant evidence	11, 12
Generalisability	21	Discuss the generalisability (external validity) of the study results	13
**Other information**			
Funding	22	Give the source of funding and the role of the funders for the present study and, if applicable, for the original study on which the present article is based	14

*Give information separately for cases and controls in case-control studies and, if applicable, for exposed and unexposed groups in cohort and cross-sectional studies.

Note: An Explanation and Elaboration article discusses each checklist item and gives methodological background and published examples of transparent reporting. The STROBE checklist is best used in conjunction with this article (freely available on the Web sites of PLoS Medicine at http://www.plosmedicine.org/, Annals of Internal Medicine at http://www.annals.org/, and Epidemiology at http://www.epidem.com/). Information on the STROBE Initiative is available at www.strobe-statement.org.

For peer review only
